# Elucidation of Natural Components of *Gardenia thunbergia* Thunb. Leaves: Effect of Methanol Extract and Rutin on Non-Alcoholic Fatty Liver Disease

**DOI:** 10.3390/molecules28020879

**Published:** 2023-01-16

**Authors:** Eman M. EL-Shial, Amal Kabbash, Mona El-Aasr, Ola A. El-Feky, Suzy A. El-Sherbeni

**Affiliations:** 1 Department of Pharmacognosy, Faculty of Pharmacy, Tanta University, Tanta 31527, Egypt; 2 Department of Pharmacognosy, Faculty of Pharmacy, Horus University, New Damietta 34518, Egypt; 3 Department of Biochemistry, Faculty of Pharmacy, Tanta University, Tanta 31527, Egypt

**Keywords:** *Gardenia thunbergia*, NAFLD, HFD, LC–ESI–MS/MS, CYP2E1, iNOS, rutin

## Abstract

The rising prevalence of non-alcoholic fatty liver disease NAFLD has strained the healthcare system. Natural products could solve this problem, so the current study focused on the impact of *G. thunbergia* Thunb. against this ailment. LC–ESI–MS/MS revealed the phytochemical profile of the methanol extract from *Gardenia thunbergia* leaves (GME). Forty-eight compounds were tentatively identified, and stigmasterol, fucosterol, ursolic acid, and rutin were isolated. The separation of the last three compounds from this plant had not before been achieved. The anti-NAFLD effect of the methanol extract of the leaves of *G. thunbergia*, and its major metabolite, rutin, was assessed in mice against high-fructose diet (HFD)-induced obesity. Male mice were allocated into nine groups: (1) saline (control), (2) 30% fructose (diseased group), (3) HFD, and 10 mg/kg of simvastatin. Groups 4–6 were administered HFD and rutin 50, 75, and 100 mg/kg. Groups (7–9) were administered HFD and methanol extract of leaves 100, 200, and 300 mg/kg. Methanol extract of *G. thunbergia* leaves at 200 mg/kg, and rutin at 75 mg/kg significantly reduced HFD-induced increments in mice weight and hepatic damage indicators (AST and ALT), steatosis, and hypertrophy. The levels of total cholesterol, LDL–C, and triglycerides in the blood decreased. In addition, the expressions of CYP2E1, JNK1, and iNOS in the diseased mice were downregulated. This study found that GME and rutin could ameliorate NAFLD in HFD-fed mice, with results comparable to simvastatin, validating *G. thunbergia’s* hepatoprotective effects.

## 1. Introduction

The active ingredients of herbal medicine and natural products have recently received increased attention as prospective therapeutic agents for the treating, and preventing of NAFLD, owing to their outstanding levels of effectiveness and minimal risks of adverse effects [1]. *Gardenia thunbergia* Thunb. (family Rubiaceae), Star Gardenia, is a shrub or small tree that thrives in Egypt and many African and Asian nations [2]. The flavonoids, terpenoids, iridoids, and steroids found in the *Gardenia* family have many therapeutic advantages, including anti-inflammatory, analgesic, and antioxidant properties, which may be attributed to flavonoids [3,4]. In the present study, LC–ESI–MS/MS was utilized to reveal, tentatively, the phytochemicals of the leaves of the plant. Recently, products from natural sources have become a substantial basis of pharmaceuticals, and they are actively being researched as potential therapeutic candidates [5]. They have a lot of benefits, including being risk-free, effective, biocompatible, and affordable [6]. Natural products, as a result, can be a successful method for preventing the emergence of various disorders, including NAFLD. 

NAFLD (non-alcoholic fatty liver disease) is a type of liver ailment that negatively impacts people’s lives. The global prevalence of NAFLD has risen to 40% [7]. NAFLD progresses from a benign condition to a threatening disease of non-alcoholic steatohepatitis, followed by pericellular fibrosis in NASH, which can lead to cirrhosis and liver cancer [7]. Steatosis, inflammation, fibrosis, cirrhosis, and, eventually, hepatocellular cancer, are all part of the pathophysiology of NAFLD. As a result, there is currently no pharmaceutical treatment for NAFLD [8,9]. Instead, almost all patients are offered food and lifestyle guidelines and instructions to increase physical activity to lose weight [10]. A treatment plan is complicated by the complexity of the pathways involved in the genesis of NAFLD. It was found that oxidative stress is a critical element in the disease’s etiology, even though the pathophysiology is complicated [11]. Multiple investigations found that a high-fat and fructose diet causes hepatic steatosis and insulin resistance in the liver sinusoidal endothelium, which is mediated, at least in part, by the activation of inducible nitric oxide synthase (iNOS) [12]. In addition, Cytochrome P450 2E1 (CYP2E1) contributes significantly to oxidative stress in NAFLD. The oxidoreductase cytochrome family causes the oxidation of different molecules, such as fatty acids, and others [13]. CYP2E1 plays a major role in NAFLD, as the levels of reactive oxygen species (ROS), such as superoxide anions, are increased after excessive fat buildup, causing oxidative injuries [13]. 

The c-Jun N-terminal kinases (c-JNKs), represent a group of protein kinases that play a vital role in stress signaling pathways that regulate cellular senescence and cell death. JNK activation has been found to be essential in modulating apoptotic signals and abnormal cell death in recent research [13]. Furthermore, recent studies have shown that overexpression of CYP2E1 increases reactive oxygen species generation causes oxidative stress and leads to persistent stimulation of c-JNK signaling pathways, which leads to hepatocyte swelling and microvascular steatosis in people with NAFLD. As a result, reducing oxidative stress and liver damage in NAFLD patients by regulating CYP2E1 and c-Junk signaling could be a helpful treatment method [13]. 

The biological activity of the methanol extract of leaves of *G. thunbergia* has only been investigated in a few research papers. For the first time, the hepatoprotective effects of the methanol extract of leaves from *G. thunbergia* (GME) was investigated in mice with NAFLD caused by a high fructose diet. The goal of this investigation was to explore whether the oral administration of GME improved hepatic steatosis in HFD-induced obesity in mice, as well as to elucidate the possible underlying molecular mechanisms and the impact of *G. thunbergia* leaves’ methanol extract on NAFLD concerning the plasma lipidomic profile changes. In addition, the aim was to evaluate the potential mechanism of action of rutin isolated from the methanol extract of *G. thunbergia* leaves. The bioactive components were tentatively recognized from the leaf extract utilizing the (–ve) ion mode of LC–ESI–MS/MS analysis. 

## 2. Results

### 2.1. LC–ESI–MS/MS Analysis of G. thunbergia Methanol Extract of Leaves (GME)

**Figure 1:**
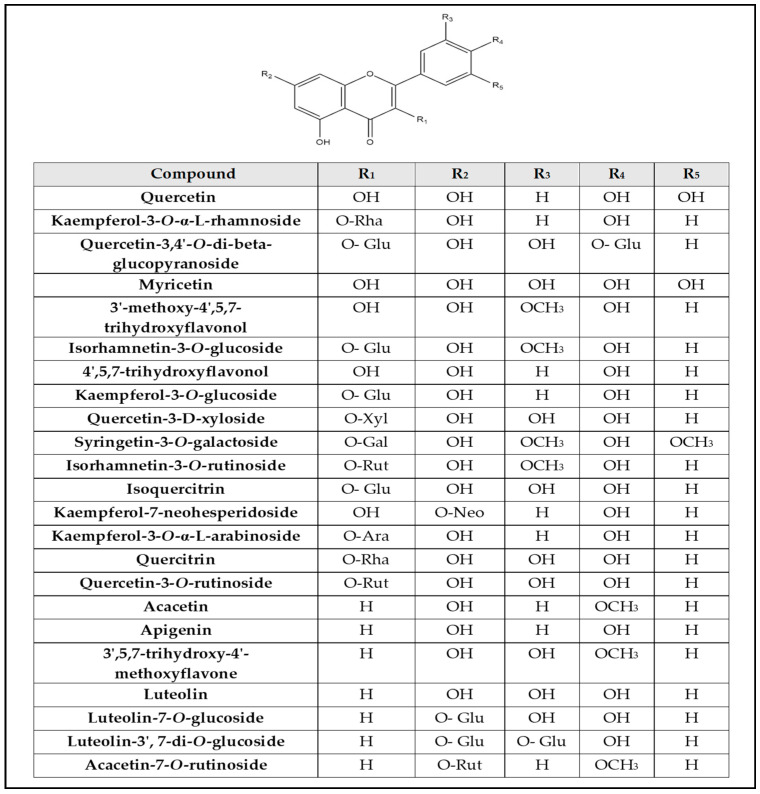
Structures of flavones, flavones glycosides, flavonols, and flavonols glycosides (Glu = glucoside, Gal = galactoside, Rha = rhamnose, Ara = arabinose, Xyl = xylose, Neo = neohesperidoside, Rut = rutinoside).

**Figure 2:**
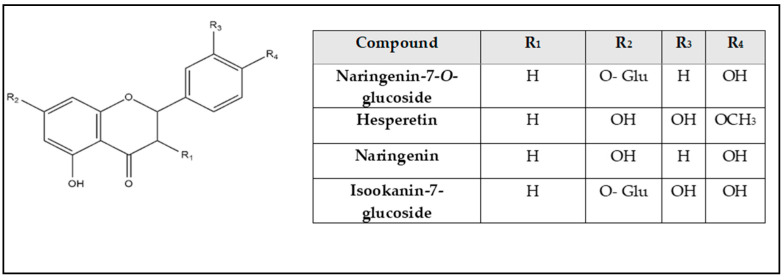
Structures of flavanones and their glycosides.

**Figure 3:**
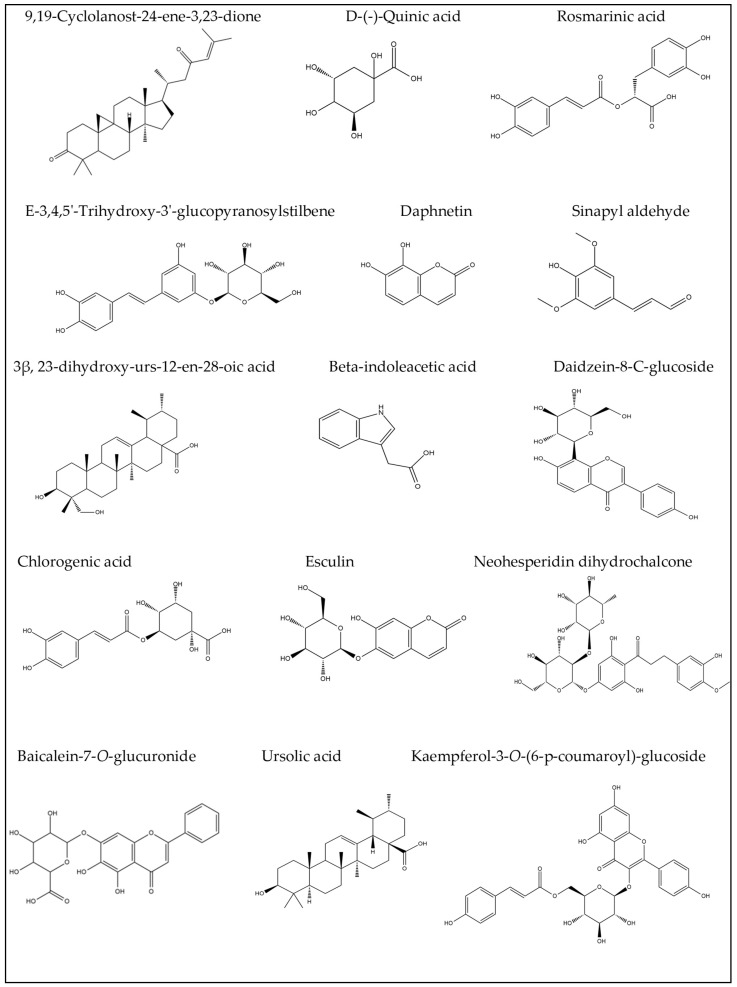
Some compounds identified tentatively of *G. thunbergia* leaves methanol extract by LC-ESI-MS/MS.

Chemical profiling of *G. thunbergia* methanol extract of leaves was tentatively recognized by liquid chromatography connected to tandem mass spectrometry of (–ve) ESI mode. Table 1 displays the existence of 48 known components of various chemical classes. The TIC is shown in Figure S3. From the Supplementary Materials, The LC–ESI–MS/MS showed different compounds of flavones, flavonols, flavanones, phenolic acids, coumaric acids and derivatives, hydroxycinnamic acids, and stilbene glycosides. In addition, flavonoids. Aglycones. and their glycosides. were recognized. The results are shown in Table 1. Structures of the recognized compounds are demonstrated in Figure 1, Figure 2 and Figure 3.

### 2.2. Identification of Compounds’ Structures Isolated from G. thunbergia Methanol Extract of Leaves

**Figure 4:**
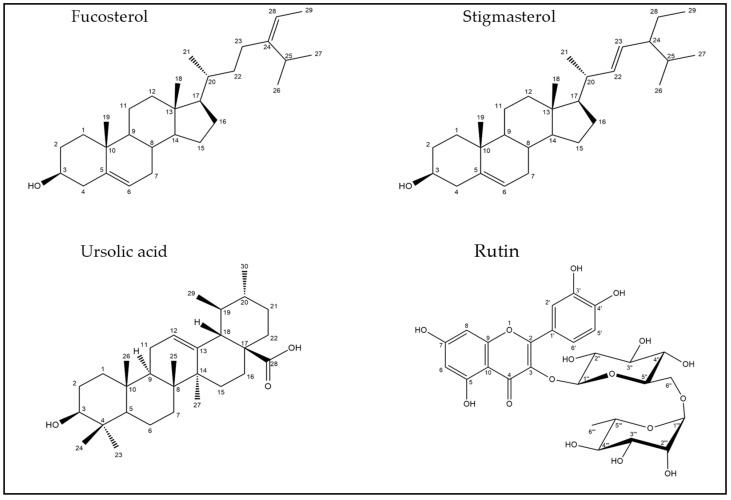
Structures of the isolated compounds from *G. thunbergia* methanol extract of leaves, (1) Fucosterol, (2) Stigmasterol, (3) Ursolic acid, and (4) Rutin.

Four compounds isolated from *G. thunbergia* methanol extract of leaves were fully elucidated by their ^1^H- and ^13^C- NMR, APT, HMBC, HSQC, and EI-MS, ESI-MS spectra. Based on their spectra, compounds 1–4 were identified as Fucosterol, Stigmasterol, Ursolic acid, and Rutin. Their structures are drawn in Figure 4.

#### 2.2.1. Spectroscopic Analysis

##### Compound (**1**): 

Amorphous white solid (24 mg), ^1^H-NMR (400 MHz, CDCl_3_), and APT-NMR (100 MHz, CDCl_3_) spectral data are displayed in the Supplementary Materials. EI-MS *m/z*: 412.18 [M]^+^. It was identified as fucosterol (stigmast-5,24-dien-3-ol) by analyzing the spectroscopic data and by comparison with the literature [40,41]. 

##### Compound (**2**): 

Amorphous white solid (33 mg), ^1^H-NMR (500 MHz, CDCl_3_), and ^13^C-NMR (125 MHz, CDCl_3_) spectral data are shown in the Supplementary Materials. EI-MS *m/z*: 412.98 [M]^+^. It was identified as stigmasterol by analyzing the spectroscopic data and by comparison with the literature [42].

##### Compound (**3**):

White amorphous powder (18 mg), ^1^H-NMR (500 MHz, DMSO), and ^13^C-NMR (125 MHz, DMSO) spectral data are found in the Supplementary Materials. EI-MS *m/z* = 456.85 [1]. It was identified as ursolic acid by analyzing the spectroscopic data and by comparison with the literature [43]. 

##### Compound (**4**): 

Yellow crystals (412 mg), ^1^H-NMR (400 MHz, DMSO), and ^13^C-NMR (100 MHz, DMSO) spectral data are found in the Supplementary Materials. ESI-MS (negative mode) [M-H]^−^ at *m/z*: 609.2. It was identified as rutin by analyzing the spectroscopic data and by comparison with the literature [44,45,46].

### 2.3. Effect of Rutin and Leaves Methanol Extract on Liver Index and Liver Function Tests of Mice

The presented data (Table 2) of the diseased group exhibited a remarkable increase in aspartate aminotransferase, alanine transaminase, and liver index, related to the control group (*p* < 0.05). Simvastatin significantly decreased the liver index and liver enzymes (*p* < 0.05) according to the diseased group. Rutin and methanolic extract of leaves displayed a remarkable decrease in ALT and AST serum levels at dose levels of rutin, 50 and 75 mg/kg, and methanol extract, 100 and 200 mg/kg (*p* < 0.05), related to the diseased group, while large doses of rutin, 100 mg/kg, and methanolic extract, 300 mg/kg, did not show significant changes according to the diseased group. The results revealed that rutin at 75 mg/kg might significantly decrease the liver index, ALT, and AST levels (*p* < 0.05) when related to the mice group administered simvastatin (10 mg/kg). Moreover, the mice group that received methanol extract at 200 mg/kg displayed a remarkable reduction in liver index related to the mice group managed with simvastatin.

#### 2.3.1. Effect of Rutin and Leaves Methanol Extract on the Lipid Profile of Mice

Table 3 demonstrates that the diseased group had high serum levels of total cholesterol, triglycerides, and low-density lipoprotein–cholesterol, while there was a noticeable decrease in the high-density lipoprotein–cholesterol of mice in the normal group (*p* < 0.05). The group administered simvastatin (10 mg/kg) exhibited a notable decrease in serum triglycerides, total cholesterol, and LDL–C and a notable increase in HDL_–_C in the diseased group (*p* < 0.05). Both rutin and methanolic extract at different doses exhibited considerable improvement in total cholesterol levels, compared to the diseased group. However, rutin at 75 mg/kg and GME at 200 mg/kg showed a reduction in triglyceride level in the diseased group (*p* < 0.05). As observed in Table 3, rutin at the dose of (50 and 100 mg/kg) and GME at (100 and 200 mg/kg) showed a notable reduction in LDL–C serum levels in the diseased group (*p* < 0.05). On the other hand, HDL–C significantly improved only at a dose of 75 mg/kg of rutin and also; improved at doses of 200 and 300 mg/kg of GME, compared to the diseased group (*p* < 0.05). 

The present data revealed that rutin at the dose of 75 mg/kg significantly improved dyslipidemia caused by the consumption of a high fructose diet, compared to the diseased group. In comparison, rutin at 50 mg/kg did not show noteworthy changes in the lipid profile of mice when compared to the diseased group. Our data demonstrated a substantial improvement of NAFLD combined dyslipidemia at 200 mg/kg of GME. On the other hand, the dose of 100 mg/kg of this extract did not show notable changes in the diseased group. 

#### 2.3.2. Effect of Rutin and Methanolic Extract on Oxidative Stress-Related Gene Expression

**Figure 5:**
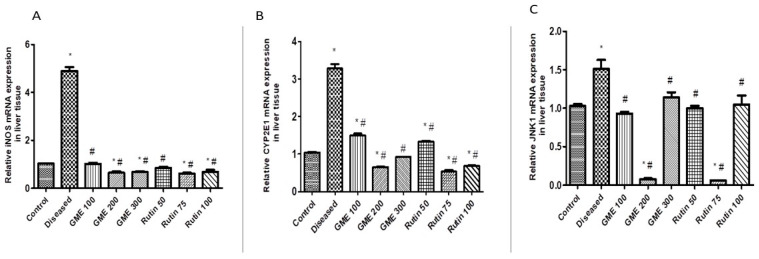
The impact of rutin and total methanolic extract at different doses on relative gene expression qRT–PCR as follows: (**A**); iNOS. (**B**); CYP2E. (**C**); JNK1. Data displayed as mean ± SD, *n* = 6 per group. Statistically, a significant difference resulted from a one-way ANOVA followed by Tukey’s multiple comparison test. Data was significant at *p*-value < 0.05. *: significant according to the normal control group. #: significant according to the diseased group. GME: *G. thunbergia* methanol extract of leaves. Control: normal control group. Diseased: diseased group.

The diseased group of mice showed a significant rise in the relative messenger ribonucleic acid expression of iNOS and CYP2E1 in the liver (*p* < 0.05) by qRT–PCR, compared to the normal control group (Figure 5A,B). It was observed that different doses of rutin and leaves methanolic extract exhibited a significant decrease in relative mRNA expression of both iNOS and CYP2E1 (*p* < 0.05), compared to the diseased group (Figure 5A,B). Interestingly, the mice group that was administered rutin at the dose of 75 mg/kg exhibited the most significant downregulation of CYP2E1 relative gene expression (decreased by 86%), relative to the diseased group. Administration of GME at 200 mg/kg and 300 mg/kg exhibited valuable downregulation in CYP2E1 relative gene expression (82% and 71%, respectively), relative to the diseased mice (Figure 5A,B).

#### 2.3.3. Effect of Rutin and Total Leaves Extract on Apoptotic Kinase (c-JNK1) Gene Expression

Figure 5C displays a notable rise in the relative mRNA expression of hepatic JNK1 of mice in the diseased group (*p* < 0.05) relative to the normal control group. It was also observed that different doses of rutin and total methanolic leaves extract exhibited a significant decrease in relative mRNA expression of JNK1 (*p* < 0.05) compared to the diseased group (Figure 5C). Furthermore, the present work displayed that the mice group taking rutin at 75 mg/kg, and the mice group receiving total *G. thunbergia* methanol extract of leaves at a dose of 200 mg/kg, exhibited the most significant downregulation of JNK1 relative gene expression (decrease by 93% and 80%, respectively) compared to the diseased mice (Figure 5C). 

#### 2.3.4. Histopathology of Liver in Different Mice Groups

**Figure 6:**
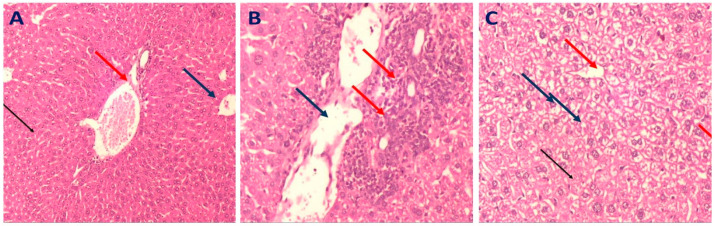
Representative photomicrograph of a liver section of mice groups (H&E, ×200) as follows: (**A**); normal control group: displayed portal tract formed of the portal venule, portal arteriole, and bile ductile (red arrows), normal sized central veins (blue arrows) surrounded by normal sized cords of hepatocytes (black arrows). (**B**); diseased group: showed central veins (red arrows) surrounded by hepatocytes with marked steatosis (blue arrows) and marked inflammation, while other cells showed individual cell necrosis. (**C**); simvastatin mice group: liver sections of mice received simvastatin (10 mg/kg) showed portal tract surrounded by mild inflammatory cellular infiltrate (red arrow) and hepatocytes with mild steatosis (blue arrow).

**Figure 7:**
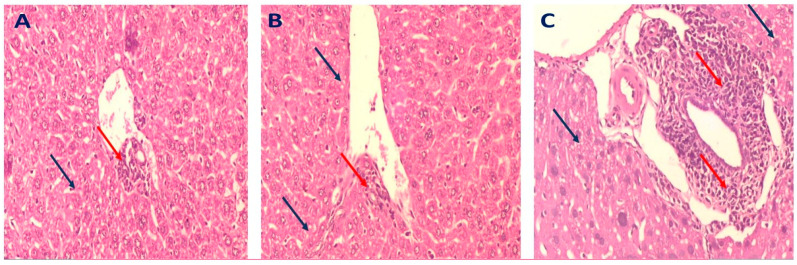
Representative photomicrograph of a liver section of mice groups that received different doses of rutin (H&E, ×200) as follows: (**A**); mice group that administered rutin at 50 mg/kg: displayed portal tract surrounded by marked inflammatory cellular infiltrate (red arrows) and hepatocytes with moderate steatosis (blue arrow). (**B**); mice group received rutin at 75 mg/kg: section of the liver showing portal tract surrounded by minimal inflammatory cellular infiltrate (red arrow) and hepatocytes with minimal steatosis and degeneration (blue arrows). (**C**); mice group that received rutin at 100 mg/kg: showed a portal tract surrounded by marked inflammatory cellular infiltrate (red arrows) surrounded by hepatocytes with moderate steatosis (blue arrow).

The liver tissues of mice in the normal group displayed a regular-sized central vein surrounded by normal hepatocytes (Figure 6A). On the contrary, the liver tissues of mice in the diseased group showed marked inflammation around the central vein and hepatocytes with marked steatosis; also, some cells exhibited necrosis (Figure 6B). Liver sections of mice that received simvastatin (10 mg/kg) revealed mild inflammatory cellular infiltrate surrounded by hepatocytes with mild steatosis (Figure 6C). Liver tissues of the mice group that were administered rutin at 75 mg/kg displayed a portal tract surrounded by minimal inflammatory cellular infiltrate surrounded by hepatocytes with minimal steatosis and degeneration (Figure 7B), relative to the marked inflammatory cellular infiltrate surrounded by hepatocytes with moderate steatosis observed in the tissues from mice in the group administered rutin at 50 mg/kg (Figure 7A). Moreover, the liver tissues from mice in the group that received rutin at 100 mg/kg displayed a portal tract surrounded by marked inflammatory cellular infiltrate surrounded by hepatocytes with moderate steatosis (Figure 7C).

**Figure 8:**
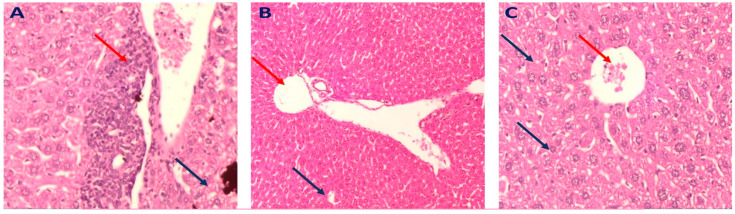
Representative photomicrograph of a liver section of mice groups that received different doses of total *G. thunbergia* methanol extract of leaves (H&E, ×200) as follows: (**A**); mice group received GME at 100 mg/kg: section of the liver displayed portal tract surrounded by marked inflammatory cellular infiltrate (red arrows) surrounded by hepatocytes with mild steatosis (blue arrow). (**B**); mice group received GME at 200 mg/kg: A section of the liver showed a portal tract (red arrow) surrounded by average-sized cords of hepatocytes (blue arrows) with no inflammation or steatosis. (**C**); mice group received GME at 300 mg/kg; showed dilated congested central vein tract (red arrow) with no inflammation and hepatocytes with mild steatosis.

The results also exhibited that the liver tissues of the mice administered GME at 200 mg/kg showed normal hepatocytes with no inflammation or steatosis (Figure 8B), when compared to the marked inflammatory cellular infiltrate surrounded by hepatocytes with mild steatosis observed in the liver tissues of mice that received GME at 100 mg/kg (Figure 8A). A mildly dilated congested central vein accompanied by mild steatosis was observed in the liver tissues of mice that received total leaves extract at 300 mg/kg (Figure 8C).

## 3. Discussion

The spread of non-alcoholic fatty liver disease is associated with increasing cases of obesity, which loads the global health care system. Due to the extraordinary consumption of high-density foods, such as high-fat or high-sugar intake diets, organ damage raises the white adipose tissue, leading to health-threatening inflammatory alterations in humans, accompanied by metabolic and hormonal changes [5].

Nutraceuticals are active phytochemical components found in plants, such as flavonoids. They are employed as a combination therapy in up to 82 percent of affected persons with fatty liver, cancer, and other disorders. Flavonoids and other phenolic compounds are essential active components among other active components [47]. The LC–ESI–MS/MS analysis of GME explored the tentative recognition of 48 known compounds contributing to various health benefits. Antioxidant properties are one of the most critical possible health benefits of phenolics (chlorogenic acid) and flavonoids, such as (rutin, hesperetin, naringenin, luteolin, kaempferol glycosides, and methoxylated flavones) [48,49]. 

Major metabolites, determined by LC–ESI–MS/MS, were rutin, isoquercitrin, delphinidin-3-*O*-(6″-*O*-α-rhamnopyranosyl-β-glucopyranoside), kaempferol-3-*O*-(6-*P*-coumaroyl)-glucoside, quinic acid, kaempferol-3-*O*-glucoside, 4′,5,7-trihydroxyflavonol, chlorogenic acid, hesperetin, acacetin, daphnetin, 3,4,5′-trihydroxy-3′-glucopyranosylstilbene, and 3,5,7-trihydroxy-4′-methoxyflavone. 

The isolation of stigmasterol, fucosterol, ursolic acid, and rutin was performed from the unsaponifiable part of the petroleum ether fraction and *n*-butanol fraction of leaves extracts. Their structures were elucidated via spectroscopic analysis of these compounds and by comparison with the literature.

Compound (1) was separated as an amorphous white matter that exhibited a molecular ion [M]^+^ peak at *m/z* 412.18 (Supplementary Materials, Figure S5). The ^1^H-NMR spectrum is provided in the Supplementary Materials, Figure S6. It showed signals resonating at δ 3.29 (d, *J* = 8 Hz, 3H, H-29), 2.30 (d, *J* = 8 Hz, 3H, H-21), 2.07 (s, 3H, H-19), 1.22 (d, *J* = 12 Hz, 3H, H-27), 0.88 (d, *J* = 8 Hz, 3H, H-26) and 0.73 (s, 3H, H-18) for six methyl groups, suggesting that compound 1 had a sterol core. The distinctive multiplet signal resonating at δ 3.47 (1H, m, H-3) was assigned to an oxygenated methine proton. In addition, there was a signal at δ 3.67 (1H, m), attributed to the proton of C-25, which appeared as a multiplet. Moreover, two olefinic proton signals displayed multiplets at δ 4.60 (1H, m, H-6) and 4.15 (1H, m, H-28), respectively. The APT-NMR data (demonstrated in the Supplementary Materials, Figure S7) explored the existence of 29 carbons (6 CH_3_ groups, 10 CH_2_ groups, 9 CH groups, and four quaternary carbons), four of which were vinylic (C-5, C-6, C-24, C-28). The signals of the APT-NMR spectrum at δ 146.36 (C-24) and 140.32 (C-5) were ascribed to two olefinic quaternary carbons, whereas two methine olefinic carbons were displayed at δ 119.63 (C-6) and 117.52 (C-28). The signal noticed at δ 71.09 ppm was attributed to a C-atom connected to an oxygen atom, which could be assigned to C-3. Carbon signals at δ 36.49 (C-20), 35.08 (C-22), 25.04 (C-23), and 34.57 (C-25) were suggested to be sp3 carbons of the aliphatic chain. This compound was identified as fucosterol (stigmast-5,24-dien-3-ol) by comparing the results with the literature [40,41].

Compound (2) exhibited [M]^+^ = 412.98 (Supplementary Materials, Figure S8). The ^1^H-NMR spectrum, displayed in the Supplementary Materials, Figure S9, showed singlet signals at δ 0.67 (3H, s) and 1.00 (3H, s), which were assigned to the presence of two methyl groups at 18 and 19 positions; three methyl doublets resonating at δ 0.81 (3H, d, *J* = 6.5 Hz), 0.83 (3H, d, *J* = 6 Hz) 0.92 (3H, d, *J* = 6.5 Hz) were assigned to carbons 26, 27 and 21, respectively; and a methyl triplet at δ 0.88 (3H) at C-29. It also displayed signals resonating at δ 5.00 (2H, m), 5.14 (2H, m), and 5.35 (1H, m), suggesting the existence of three protons, according with those of a trisubstituted and a disubstituted olefinic bond at C-22, C-23, and C-6 respectively. The proton ascribed to the H-3 of a sterol moiety appeared as a multiplet signal at δ 3.51; this characteristic signal was assigned to an oxygenated methine proton. The ^13^C- NMR spectral data (demonstrated in the Supplementary Materials, Figure S10) reinforced the presence of 29 carbon signals, including six methyls and nine methylenes. The chemical shift at δ (C-3) 71.80 was due to oxymethine carbon, and signals at δ C 140.73 and δ C 121.72 were due to a double bond between C-5 and C-6, respectively. Carbon signals at δ C 138.32 (C-22) and δ C 129.24 (C-23) were ascribed to carbon in a trans-conformation of a triterpenoid nucleus. The structure was elucidated as stigmasterol, by analyzing the spectroscopic data in the literature [42,50].

The EI–MS spectrum of compound (3) had [M]^+^ at *m/z* 456.85. The ^1^H–NMR spectrum of the compound displayed the existence of seven methyl groups resonating at δ 0.71 (3H, s, H-25), 0.82 (3H, s, H-24), 1.00 (3H, s, H-26), 1.05 (3H, s, H-27), 1.19 (3H, s, H-23) and 0.76 (3H, d, *J* = 5.75 Hz, H-29), 0.86 (3H, d, *J* = 6.7 Hz, H-30) in the area of the higher field. which is common in an ursane-type triterpenes skeleton [50]. A down-field signal at δ 2.97 (1H, m) represented H-3, which was assigned to the methine proton on the carbon, which bore the hydroxyl group (its proton signal was displayed at δ 4.30), and an olefinic proton signal resonating at δ 5.09 (1H, s) ppm was assigned to H-12 of a triterpene. Finally, a doublet signal resonated at δ 2.46 (1H, d, *J* = 11.3 Hz) and was assigned to C-18. The ^13^C- NMR spectrum indicated that compound (3) had a total of 30 carbon atoms and showed the presence of signals characteristic to the triterpene group of α-amyrine: δ_C_ 77.44, 125.10, 138.70, and 178.91 ppm. The signal resonating at δ 77.44 ppm was ascribed to C-3 (CH–O–) of a triterpenoid and the signals at δ 125.10 and 138.70 ppm at position 12 and 13, respectively, were for the double bond of ursane type of triterpenes [28]. The signal resonating at δ_C_ 178.91 ppm was attributed to a carbon of a carbonyl group at position 28. It was identified as ursolic acid, according to the reported data in the literature [43]. Different spectra are provided in the Supplementary Materials, Figures S11–S13.

The ESI–MS spectrum of compound (4) in the negative ion mode was demonstrated [M-H]^−^ at *m/z* 609.2. The ^1^H–NMR spectrum showed aliphatic signals assigned to the sugar part, as two hexoses were displayed at δ 5.35 (d, *J* = 6.8 Hz, H-glc-1, anomeric proton), 3.37 (m, H-glc-2), 3.73 (d, *J* = 10.4 Hz, H-glc-6), 4.39 (s, H-rha-1, anomeric proton), 3.37 (m, H-rha-2), 3.24 (m, H-rha-3), 3.37 (m, H-rha-4), 3.24 (m, H-rha-5), 1.01 (d, *J* = 6 Hz, H-rha-6). The doublet signal at δ 5.35 ppm (*J* = 6.8 Hz) was assigned to the anomeric proton and confirmed the *β*-configuration of glucose. The presence of a doublet signal resonating at δ 1.01 ppm with *J* = 6 Hz for the methyl group and a singlet signal resonating at δ 4.39 ppm for the acetal proton were attributed to rhamnose. The ^1^H–NMR spectrum showed meta-coupled doublets resonating at δ 6.20 (d, *J* = 2 Hz) and 6.40 (d, *J* = 1.2 Hz) ppm, each ascribed to 1 proton of H-6 and H-8, respectively. The 3′, 4′ dihydroxylation on ring B was detected by the presence of signals at δ 7.54 and 7.56 ppm, which were ascribed to H-2′, 6′. The notable signal was presented at δ 6.84 (d, *J* = 8.4 Hz), representing the proton of H-5′ on ring A. The ^13^C–NMR spectrum indicated that compound (4) had a total of 27 carbon atoms: carbon signals at δ 101.63 (C-glc-1), 74.52 (glc-2), 76.88 (glc-3), 68.71 (glc-4), 76.35 (glc-5), 67.45 (glc-6), confirmed the presence of glucose moiety, and carbon signals at δ101.21 (C-rha-1), 71.00 (rha-2), 70.44 (rha-3), 70.82 (rha-4), 72.28 (rha-5), 18.21 (rha-6), were attributed to the presence of rhamnose moiety. The up-field signal resonating at δ 18.21 ppm of (rha-6) was noticed with signals for the dihydroxy groups at 3`, 4` of ring B. Signals which were ascribed at δ 101.63 and 101.21 ppm, were attributed to acetal carbon of (C-glc-1) and (C-rha-1). A recognized down-field signal showed at δ 177.81 ppm for C-4. The spectrum also showed different down-field signals resonating at δ 164.65, 157.06, 161.66, and 156.88 ppm for C-7, 9, 5, and 2, respectively. Correlation between carbons and protons displayed the proton-carbon coupling with nearest carbon skeletons and was confirmed by the Heteronuclear Multiple Bond Correlation spectrum, as shown in the Supplementary Materials, Figure S18. It was identified according to the reported data in the literature [44,45,46]. Different spectra were found in the Supplementary Materials, Figures S15–S17.

In the present work, induction of NAFLD, mediated by administration of HFD in drinking water, was confirmed by the significant increase in the liver index and elevated serum levels of AST, ALT, TG, TC, LDL-C, and the low concentration of HDL–C. Histopathological examination of mice from the disease control group showed marked inflammation around the central vein and hepatocytes with marked steatosis, confirming the induction of NAFLD, as previously described by Zhang et al. [51]. Furthermore, the significantly increased levels of triglyceride and total cholesterol in mice of the disease control group indicated fat deposition in the liver tissue. 

Both rutin and methanol extract of leaves of *Gardenia thunbergia* showed notable improvement in the lipid profile of treated mice groups, compared to the diseased group. Interestingly, rutin at the dose of 75 mg/kg and GME at 200 mg/kg were the doses that displayed a significant decrease in triglyceride levels, compared to the diseased group. Rutin at (50 and 100 mg/kg) and GME at (100 and 200 mg/kg) showed a notable reduction in LDL–C serum levels, compared to the diseased group. HDL–C improved only at a dose of 75 mg/kg of rutin and 200 and 300 mg/kg of GME, compared to the diseased group.

These results indicated that administration of GME or pure rutin exhibited a decrease in fat accumulation in hepatocytes, resulting in the decreased inflammation and steatosis observed in liver sections of mice from the treated groups. 

The reducing effect of GME on AST and ALT levels indicated that it might reduce the adverse effects of a high fructose diet on the liver and lessen hepatic injury, which agreed with Li et al. [49], who demonstrated that flavonoids had a positive effect on liver structure and function.

The inflammatory response plays an essential role in developing hepatic steatosis [52]. Accumulation of fatty acids could be handled by cytochrome (CYP2E1), a member of the oxidoreductase cytochrome family [53,54,55]. However, increased CYP2E1 expression contributes considerably to oxidative stress in NAFLD livers according to various studies, including human and mouse models of NAFLD [56,57,58]. In addition, CYP2E1 triggers hydroxylation of fatty acids, resulting in the accumulation of cytotoxic lipid intermediates and ROS and activating of cell death by activating the ERK1/2 pathway [56].

It was found that upregulation of CYP2E1 played a potential role in the activation of the death signaling c-Jun N-terminal kinases (cJNK) pathway [59,60,61]. Activation of cJNK would, consequently, phosphorylate and activate other proteins, such as Bax and Bcl-2, which would, finally, trigger mitochondrial apoptotic pathways and subsequent development and progression of NAFLD. Increased steatosis and fatty acid β-oxidation would stimulate reactive oxygen species and oxidative stress, leading to increased cJNK activity, and, subsequently, increased insulin resistance, provoking hepatocyte death [59,60,61]. 

These results indicated that flavonoids of *G. thunbergia,* especially rutin, could hinder the development and progression of NAFLD through the downregulation of CYP2E1, which, subsequently, inhibits the c-JNK apoptotic pathway. Histopathological examination of liver tissues of the mice groups administrated rutin (at the dose of 75 mg/kg) or GME (at the dose of 200 mg/kg) revealed the absence of inflammation and steatosis, which was considered as further evidence for the potential role of *G. thunbergia* in inhibiting NAFLD development. Our results were consistent with other studies that reported that TNF-α, FFAs, and ROS activated JNK and promoted initiation and progression of insulin resistance and the development of NAFLD [62,63].

GME and rutin also reduced iNOS expression levels. Free-radical scavenging activity of GME polyphenolics could decrease ROS and prevent inflammatory reactions. These results were consistent with Li et al. [49], who reported that the downregulation of iNOS inhibited the development of insulin resistance in mice fed HFD. 

Further assessment of liver tissues of mice given GME (200 mg/kg) displayed a portal tract surrounded by average-sized cords of hepatocytes with no inflammation or steatosis. Mice treated with rutin (75 mg/kg) showed a portal tract surrounded by minimal inflammatory cellular infiltrate and hepatocytes with minimal steatosis and degeneration. 

GME (300 mg/kg) showed a dilated congested central vein tract (red arrow) with no inflammation and hepatocytes with mild steatosis. Mice treated with rutin (100 mg/kg) revealed marked inflammatory cellular infiltrate surrounded by hepatocytes with mild steatosis and a congested central vein tract, compared to normal hepatocytes, and the absence of steatosis or inflammation was observed in mice given rutin at the dose of (75 mg/kg) or GME (200 mg/kg). 

Administration of *G. thunbergia* extract of leaves at a dose of (200 mg/kg) and rutin (75 mg/kg) could prevent the development of NAFLD in HFD-fed mice through decreasing the oxidative stress mediated by β-oxidation of fatty acids, and decreasing inflammatory steatosis development through downregulation of the CYP2E1–cJNK apoptotic signaling pathway.

## 4. Materials and Methods

### 4.1. Plant Material

*Gardenia thunbergia* Thunb. Leaves were obtained from the shrubs growing in Al Sadat City, Egypt, in March, 2018. Professor Dr. Ibrahim Abd El Rahim Mashaly, Professor of Plant Ecology and Flora, Department of Botany, Faculty of Science, Mansoura University, certified the plant’s legitimacy. The leaves were air-dried and then ground before use for this study. The voucher specimen (PG00415-M) was kept at the Herbarium of the Department of Pharmacognosy, Faculty of Pharmacy, Tanta University.

#### Extraction and Isolation Procedure

*Gardenia thunbergia* leaves were dried at room temperature and ground to obtain 1.5 kg dry powder. They were extracted by cold maceration with methanol (3 × 3 L, 48 h each) till exhaustion and then evaporated using a rotary evaporator under reduced pressure at 40 °C to provide a dry leaves methanol extract of (220 g) yield. A part of the methanol extract (175 g) was resuspended in methanol and successively partitioned with petroleum ether 60–80, methylene chloride, ethyl acetate, and *n*-butanol. The yield of each fraction was petroleum ether (45 g), methylene chloride (40.1 g), ethyl acetate (4.6 g), and *n*-butanol (55.6), represented in the Supplementary Materials, Figure S1.

The petroleum ether fraction obtained from the methanolic extract of the leaves was used to investigate the saponifiable and unsaponifiable matter. Two grams of petroleum ether extract of the methanol extract of the leaves was saponified by heating for 6 h using a boiling water bath with a reflux condenser, accompanied by 30 mL alcoholic KOH (10%). To eliminate the majority of the alcohol, the saponified liquid was distilled, then the aqueous liquid was diluted with 20 mL water before being extracted with ether till exhaustion. The combined ethereal extracts were washed with distilled water, dehydrated over anhydrous Na_2_SO_4_, and distilled under a vacuum to yield the unsaponifiable matter (USM) of *G. thunbergia* methanol extract of leaves, represented in the Supplementary Materials, Figure S2. The unsaponifiable matter of the petroleum ether fraction (1 g) was applied to a silica gel column (40 g, φ two × 30 cm, fraction collected 30 mL), using the wet method. The mobile phase was composed of gradient mixtures with increasing polarities of petroleum ether—CH_2_Cl_2,_ then methylene chloride: methanol mixtures of increasing polarities. The petroleum ether—CH_2_Cl_2_ (98:2) afforded compound (1) (24 mg). The petroleum ether—CH_2_Cl_2_ (30:70) eluate (100 mg) was applied to another CC, using silica gel (6 g, φ one × 6 cm, fraction collected 10 mL), using a mobile phase of increasing polarity (gradient elution) of *n*-Hexane—EtOAc. The *n*-Hexane—EtOAc (10:90) afforded compound (2) (33 mg). The first column afforded eluate (82 mg) by methylene chloride—methanol (98:2) was applied to another CC using silica gel (6 g, φ 1 × 6 cm, fraction collected 10 mL). Elution was done successively with a mixture of increasing polarities of CH_2_Cl_2_—MeOH to give compound (3) (18 mg) at (93:7), displayed in Figure S4 of the Supplementary Materials.

The *n*-butanol fraction (55.6 g) was mixed with deionized water and the suspension was passed through a Diaion HP-20 column [60 × 3 cm, 80 g]. The mobile phase was composed of deionized water (1 L), followed by MeOH-H_2_O (50:50) and 100% MeOH. Next, the *n*-butanol fraction, eluted with 100% MeOH (5.5 g), was applied to a column of silica gel (200 g, φ 4.5 × 40 cm, fractions of 50 mL each) using the dry method and eluted, consecutively, with a mixture of increasing polarities of CH_2_Cl_2_—MeOH containing 5% water. A yellow matter was obtained from the CH_2_Cl_2_—MeOH containing 5% water (70:30) eluate (435 mg). This matter was applied to Sephadex LH-20 for purification to obtain a compound (4) (412 mg), represented in the Supplementary Materials, Figure S14.

### 4.2. LC–ESI–MS/MS for Metabolite Analysis

#### 4.2.1. Sample Preparation

The sample was prepared by mixing the powder from *Gardenia thunbergia* leaves with light petroleum ether. The extraction of the active constituents of the powder with MeOH was done after exhaustion with petroleum ether; then, the extract was concentrated under a vacuum at 40 ^◦^C. The dried matter (50 mg) was then subjected to the procedure previously adopted, mentioned in the literature [64], before analysis. In addition, the LC–ESI–MS/MS analysis method was mentioned in the literature [64].

#### 4.2.2. Acquisition Method and Analytical Parameters

Actives in the extract were identified by the Proteomics and Metabolomics Unit, Children’s Cancer Hospital (57357), Basic Research Department, Cairo, Egypt. The methods used were adopted from previously reported literature [65,66,67].

### 4.3. Experimental Animals

Male Albino mice, five-week-old, weight 20 g, were bought from the animal house of Giza Institute of Ophthalmology, Cairo, Egypt. The animals were retained at 25 ± 2 °C in a 12 h light-dark cycle. All mice were permitted to eat and drink freely.

#### 4.3.1. Method of Assessment of the Effect of Methanol Extract and Rutin on Non-Alcoholic Fatty Liver Disease

After the acclimatization period (1 week), mice were separated into nine groups randomly (6 mice for each group). Then, they were treated with different drugs as follows: 

Group 1: Normal control (NC): administered the diet and water freely for eight weeks. 

Group 2: Diseased group (DG): administered 1 mL of 0.9% normal saline orally along with the diet and fructose in drinking water. 

Group 3: Simvastatin (HFD/Simvastatin): administered simvastatin (10 mg/kg) by oral gavages.

Group 4: Rutin 50 mg/kg (HFD/LDR): orally administered rutin (50 mg/kg) through oral gavages.

Group 5: Rutin 75 mg/kg (HFD/MDR): orally administered rutin (75 mg/kg) through oral gavages.

Group 6: Rutin 100 mg/kg (HFD/HDR): orally administered rutin (100 mg/kg) through oral gavages. 

Group 7: Total leave extract 100 mg/kg (HFD/LDGME): administered total leave extract (100 mg/kg) by oral gavages.

Group 8: Total leave extract 200 mg/kg (HFD/MDGME): administered total leave extract (200 mg/kg) by oral gavages.

Group 9: Total leave extract 300 mg/kg (HFD/HDGME): administered total leave extract (300 mg/kg) by oral gavages.

The induction of NAFLD in mice was performed using 30% fructose in drinking water [68]. All groups were administered the diet and solution of fructose (30%) in drinking water free from the 2nd–8th week. The dose of simvastatin was administered twice weekly for seven weeks. In addition, groups 4–6 were administered the doses of rutin twice weekly. Also, groups (7–9) were administered the doses of methanol extracts twice weekly. Food and drinking water intake were monitored weekly.

HFD: high-fructose diet, LDR: Low dose rutin, MDR: Moderate dose rutin, HDR: High dose rutin, LDGME: low dose gardenia methanol extract, MDGME: Moderate dose gardenia methanol extract, HDGME: high dose gardenia methanol extract.

#### 4.3.2. Determination of Liver Index and Liver Function Tests

Fresh liver samples were dissected after the scarification of mice. The determination of liver index, aspartate aminotransferase (AST), and alanine aminotransferase (ALT) levels in serum samples [69] were performed using colorimetric kits purchased from Spectrum-diagnostics^®^, Cairo, Egypt.

#### 4.3.3. Determination of Lipid Profile in the Serum

At the end of the experiment (8 weeks), mice were kept for overnight fasting. Then, mice of all groups were anesthetized and sacrificed by cervical dislocation, and aliquots of mice serum samples were collected and stored at ࢤ20 °C for biochemical analysis. Kits for determining serum biomarkers of lipid profile, adopting manufacturer protocol [70,71], were purchased from Bio-diagnostic^®^, Cairo, Egypt. 

#### 4.3.4. Quantitative Real-Time Polymerase Chain Reaction (qRT-PCR) for Liver Tissue

The liver tissue samples of mice were homogenized to determine iNOS, CYP2E1, and c-JNK1 relative gene expression. A total RNA extraction kit (Bio E-Technology, Xi’an, China) was used to extract total RNA from the liver tissue of mice; purity and concentration of mRNA were estimated. The extracted mRNA was used to form cDNA. Primer sequences were mentioned in the Supplementary Materials. Each sample was analyzed by the qRT-PCR, normalized to the reference gene level (GAPDH) level, and expressed as a relative copy number (RCN). Threshold cycle (Ct) values were calculated, and the transcript levels were calculated by using 2^−ΔCt^ formula [68].

#### 4.3.5. Histopathological Examination of Liver Tissue

Liver tissue was cut into five μm sections, which were kept in paraffin, hematoxylin, and eosin were used in staining. A professional histopathologist examined these sections with a light microscope (Leica DM 500, Switzerland). The liver sections were investigated for inflammatory cell infiltration, necrosis, and the areas scored steatosis [72].

### 4.4. Statistical Analysis

Statistical analysis was performed with the statistical package for social science (SPSS) software version 20. One-Way ANOVA test was done for one-independent variables. The least significant difference (LSD) test is used in the analysis of variance for multiple comparisons in studying liver index, liver function tests, and the lipid profile of mice. Data displayed as mean ± SD, *n* = 6 per group. Statistically, a significant difference was determined in the qRT-PCR experiment by a one-way analysis of variance followed by Tukey’s multiple comparison test. Data was significant at *p*-value < 0.05. 

## 5. Conclusions

The findings of this study indicated that *G. thunbergia* methanol extract of leaves and rutin isolated from this plant had a distinguishable effect in reducing lipid serum levels. Fucosterol, ursolic acid, rutin, and stigmasterol were isolated from the leaves. The first three actives were separated for the first time from this species. Different compounds (48) from several phytochemical classes were tentatively identified using LC–ESI–MS/MS to explore the chemical composition of this plant. The presence of the major metabolites of rutin, isoquercitrin, delphinidin rhamnoglucoside, kaempferol glycosides, quinic acid, 4′,5,7-trihydroxyflavonol, chlorogenic acid, hesperetin, acacetin, daphnetin, 3,4,5′-trihydroxy-3′-glucopyranosylstilbene, and 3,5,7-trihydroxy-4′-methoxyflavone with anti-inflammatory, and antioxidant properties, could explain the therapeutic effects of *G. thunbergia* methanol extract of leaves in the non-alcoholic fatty liver disease (NAFLD). *G. thunbergia* extract and rutin could hinder the development of NAFLD by reducing oxidative stress and triggering the downregulation of CYP2E1 and c-JNK cell death pathway. Therefore, *G. thunbergia* extract and rutin could be potential therapeutic options for treating NAFLD. However, further clinical studies are required to assess potentially toxic and effective doses. 

## Figures and Tables

**Table 1:** Phytochemical profiling of *G. thunbergia* methanol extract of leaves by LC–ESI–MS/MS in negative ion mode.

No.	Rt (min.)	[M − H]¯ *m/z*	Error PPM	Matching Score	MS^2^ *m/z*	Formula	Identification	Ref
1	1.05	178.969	9.1	76.1	179.054, 161.043	C_9_H_8_O_4_	Caffeic acid	[6]
2	1.07	507.049	−0.2	72.7	507.050, 444.951	C_23_H_24_O_13_	Syringetin-3-*O*-galactoside	[13]
3	1.08	317.050	1.1	77	317.056, 273.071, 249.004, 213.035, 191.055, 80.963	C_15_H_10_O_8_	Myricetin	[14]
4	1.08	405.098	2.5	71.9	405.099, 404.874, 299.083, 191.055	C_20_H_22_O_9_	E-3,4,5′-Trihydroxy-3′-glucopyranosylstilbene (Astringin)	[15]
5	1.10	191.055	2.6	85.2	191.055, 190.445, 173.040, 171.028, 153.020, 137.023	C_7_H_12_O_6_	D-(-)-Quinic acid	[16]
6	1.16	174.077	−0.3	81.9	174.074, 144.068, 128.066	C_10_H_9_NO_2_	*β*-indoleacetic acid	[17]
7	1.24	447.112	−0.9	86.3	447.077, 446.996, 379.080, 191.053, 102.954	C_21_H_20_O_11_	Quercitrin	[18]
8	1.29	163.06	−3.3	86.2	163.060, 119.041	C_9_H_8_O_3_	3-(4-hydroxyphenyl)prop-2-enoic acid	[19]
9	1.45	593.153	−2.4	82.5	593.112, 285.301, 248.955	C_27_H_30_O_15_	Kaempferol-7-neohesperidoside	[6]
10	1.81	353.088	−0.7	86.1	353.065, 191.055, 179.037, 161.023	C_16_H_18_O_9_	Chlorogenic acid	[20]
11	3.70	339.104	−21.9	88.1	339.112, 177.014	C_15_H_16_O_9_	Esculin	[21]
12	4.14	445.135	−0.4	81.1	445.135, 269.425, 161.032	C_21_H_18_O_11_	Baicalein-7-*O*-glucuronide	[22,23]
13	5.09	315.108	1	77.1	315.108, 269.098	C_16_H_12_O_7_	3′-methoxy-4′,5,7- trihydroxyflavonol (Isorhamnetin)	[6]
14	5.44	431.189	4.1	84.2	431.189, 385.187, 335.081, 285.524, 223.133, 205.128	C_21_H_20_O_10_	Kaempferol-3-*O*-*α*-L-rhamnoside (Afzelin)	[6]
15	6.24	609.174	−46	91.9	609.143, 607.169, 343.025, 301.033, 300.027, 299.059, 272.036	C_27_H_31_O_16_	Quercetin-3-*O*-rutinoside (Rutin)	[22]
16	6.25	609.143	3.1	92.1	609.143, 607.169, 343.025, 302.415, 301.033, 300.027, 299.059	C_27_H_31_O_16_	Delphinidin-3-*O*-(6″-*O*-*α*-rhamnopyranosyl-*β*-glucopyranoside)	[24]
17	6.58	463.088	−0.9	92.7	463.085, 461.594, 445.094, 343.051, 301.038, 300.026	C_21_H_20_O_12_	Isoquercitrin	[25]
18	6.78	449.104	−6.2	86.4	449.111, 287.040, 151.006	C_21_H_22_O_11_	Isookanin-7-glucoside	[26]
19	6.86	593.152	−1	91.3	593.150, 591.226, 285.039, 284.005, 255.031	C_30_H_26_O_13_	Kaempferol-3-*O*-(6-p-coumaroyl)-glucoside	[6]
20	6.88	433.076	−0.2	84.9	433.056, 301.078	C_20_H_18_O_11_	Quercetin-3-D-xyloside	[27]
21	6.99	623.162	−0.6	85.5	623.155, 315.036	C_28_H_32_O_16_	Isorhamnetin-3-*O*-rutinoside	[28]
22	7.00	447.095	−3.9	85.1	447.093, 285.043, 284.031, 255.028	C_21_H_20_O_11_	Luteolin-7-*O*-glucoside	[20]
23	7.2	447.092	0.2	87	447.096, 445.646, 285.038, 284.033, 256.033, 255.033	C_21_H_20_O_11_	Kaempferol-3-*O*-glucoside (astragalin)	[29]
24	7.30	415.197	−0.6	77.6	415.189, 392.128, 253.524, 179.055, 170.951	C_21_H_20_O_9_	Daidzein-8-C-glucoside (Puerarin)	[30]
25	7.46	477.106	−2.5	80.8	477.097, 409.160, 315.530	C_22_H_22_O_12_	Isorhamnetin-3-*O*-glucoside	[28]
26	7.68	433.111	3.4	81.4	433.118, 387.207, 271.056	C_21_H_22_O_10_	Naringenin-7-*O*-glucoside	[31]
27	8.70	193.049	−3.3	86.2	193.050, 178.030, 162.036, 161.022	C_10_H_10_O_4_	3-(4-hydroxy-3-methoxyphenyl) prop-2-enoic acid	[32]
28	9.51	301.032	9.1	79.4	301.035, 178.998, 150.994	C_15_H_10_O_7_	Quercetin	[28]
29	9.55	359.170	1.6	71.2	359.132, 331.070	C_18_H_16_O_8_	Rosmarinic acid	[21]
30	9.79	417.156	−38.6	83.5	417.160, 285.412	C_20_H_18_O_10_	Kaempferol-3-*O*-*α*-L-arabinoside	[31]
31	9.89	625.153	1.4	80.5	625.148, 579.297, 301.634	C_27_H_30_O_17_	Quercetin-3,4′-*O*-di-*β*-glucopyranoside (QDG)	[32]
32	10.25	271.061	0.3	80.7	271.063, 252.033	C_15_H_12_O_5_	Naringenin	[24]
33	10.38	177.054	2.6	73.3	177.055, 162.021, 145.030, 134.038, 121.022, 118.041	C_9_H_6_O_4_	Daphnetin	[33]
34	10.60	609.160	−23.6	85.4	609.149, 285.152	C_27_H_30_O_16_	Luteolin-3′,7-di-*O*-glucoside	[34,35]
35	10.76	207.065	0.7	89	207.066, 192.041, 177.025, 133.028	C_11_H_12_O_4_	Sinapyl aldehyde	[36]
36	10.81	285.040	0.8	83	285.036	C_15_H_10_O_6_	Luteolin	[28]
37	12.31	301.072	0.8	71.8	301.072, 165.008, 135.044, 134.036	C_16_H_14_O_6_	Hesperetin	[6]
38	13.57	269.080	2.6	85.4	269.079	C_15_H_10_O_5_	Apigenin	[28]
39	13.80	285.076	1	77.1	285.076, 191.036, 165.014, 119.041	C_15_H_10_O_6_	4′,5,7-trihydroxyflavonol	[6]
40	14.00	283.062	−2.4	84.9	283.060, 282.158, 268.037, 240.039, 149.996	C_16_H_12_O_5_	Acacetin	[6]
41	14.20	299.055	0.9	74.2	299.056, 284.032, 271.044, 256.030, 243.068, 240.032	C_16_H_12_O_6_	3′,5,7-trihydroxy-4’-methoxyflavone	[6]
42	14.40	591.197	−35.1	86.6	591.174, 283.065	C_28_H_32_O_14_	Acacetin-7-*O*-rutinoside (Linarin)	[31]
43	16.10	471.347	0	77.9	471.345	C_30_H_48_O_4_	3*β*,23-dihydroxy-urs-12-en-28-oic acid	[37]
44	16.87	469.328	6	79.3	469.329	C_30_H_46_O_4_	9,19-Cyclolanost-24-ene-3,23-dione	[25]
45	21.33	279.233	−0.9	81.8	279.234	C_18_H_32_O_2_	9–12-Octadecadienoic acid	[38]
46	22.63	455.353	-0.9	79.3	455.320	C_30_H_48_O_3_	Ursolic acid	[25]
47	23.12	255.233	−0.4	83	255.231	C_16_H_32_O_2_	Palmitic acid	[38,39]
48	24.00	611.154	−3.4	84.3	611.147	C_28_H_36_O_15_	Neohesperidin dihydrochalcone	[39]

**Table 2:** Impact of rutin and leaves methanol extract on liver index and liver function tests of mice.

Groups	Liver Index (%)	ALT (U/L)	AST (U/L)
Control (NC)	3.50 ± 0.76	21.59 ± 0.49	25.96 ± 0.51
Diseased group (DG)	4.84 ± 0.86 ^a^	110.25 ± 0.86 ^a^	86.91 ± 0.73 ^a^
Simvastatin	3.66 ± 0.64 ^b^	76.98 ± 0.66 ^b^	76.89 ± 0.66 ^b^
Rutin 50 mg/kg	4.76 ± 0.79	89.63 ± 0.74 ^b^	79.34 ± 0.67 ^b^
Rutin 75 mg/kg	2.71 ± 0.63 ^b,c^	60.71 ± 0.51 ^b,c^	71.29 ± 0.66 ^b,c^
Rutin 100 mg/kg	4.21 ± 0.69	102.30 ± 0.79 ^b^	84.36 ± 0.71 ^b^
*G. thunbergia* methanol extract of leaves 100 mg/kg	3.92 ± 0.51 ^b^	95.84 ± 0.69 ^b^	80.99 ± 0.61 ^b^
*G. thunbergia* methanol extract of leaves 200 mg/kg	2.85 ± 0.53 ^b,c^	80.36 ± 0.69 ^b^	78.63 ± 0.65 ^b^
*G. thunbergia* methanol extract of leaves 300 mg/kg	4.76 ± 0.73	110.60 ± 0.88	86.01 ± 0.63

Data displayed as mean ± SD, *n* = 6 per group. NC: normal control, DG: diseased group. Statistics were calculated by one-way ANOVA followed by an LSD comparison test. ^a^: significant according to the normal control group, ^b^: significant according to the diseased group, ^c^: significant according to the simvastatin group. Significance was assessed at *p* < 0.05.

**Table 3:** Effect of rutin and leaves methanol extract on the lipid profile of mice.

Groups	TC (mmole/L)	TG (mmole/L)	HDL-C (mmole/L)	LDL-C (mmole/L)
NC	2.55 ± 0.56	1.56 ± 0.44	2.11 ± 0.66	2.09 ± 0.76
DG	10.40 ± 0.74 ^a^	2.65 ± 0.71 ^a^	1.36 ± 0.33 ^a^	4.96 ± 0.88 ^a^
Simvastatin	3.97 ± 0.41 ^b^	1.58 ± 0.66 ^b^	2.25 ± 0.52 ^b^	2.11 ± 0.51 ^b^
Rutin 50 mg/kg	6.84 ± 0.64 ^b^	2.01 ± 0.37	1.50 ± 0.43	4.80 ± 0.86
Rutin 75 mg/kg	4.36 ± 0.48 ^b^	1.66 ± 0.58 ^b^	2.34 ± 0.48 ^b^	2.99 ± 0.66 ^b^
Rutin 100 mg/kg	7.90 ± 0.76 ^b^	2.33 ± 0.76	1.53 ± 0.36	3.76 ± 0.41 ^b^
*G. thunbergia* methanol extract of leaves 100/kg	5.33 ± 0.61 ^b^	2.05 ± 0.86	1.62 ± 0.35	3.99 ± 0.43 ^b^
*G. thunbergia* methanol extract of leaves 200/kg	4.97 ± 0.53 ^b^	1.69 ± 0.74 ^b^	2.30 ± 0.66 ^b^	3.01 ± 0.56 ^b^
*G. thunbergia* methanol extract of leaves 300/kg	6.01 ± 0.71 ^b^	2.31 ± 0.85	2.40 ± 0.57 ^b^	4.76 ± 0.81

Data presented Mean ± SD, *n* = 6 per group. NC: normal control, DG: diseased group. *p* < 0.05 was significant by one-way ANOVA followed by LSD comparison test. ^a^: significant according to the normal control group, ^b^: significant according to the diseased group.

## Data Availability

All the data supporting results were included in the Supplementary Materials.

## Supplementary Materials

The following supporting information can be downloaded at: https://www.mdpi.com/article/10.3390/molecules28020879/s1, Figure S1: Extraction and fractionation of *G. thunbergia* methanol extract of leaves; Figure S2: Investigation of petroleum ether extract of *G. thunbergia* methanol extract of leaves; Figure S3: The total ion chromatogram (TIC) of *G. thunbergia* methanol extract of leaves (negative mode); Figure S4: Investigation of the unsaponifiable matter (USM) of *G. thunbergia* methanol extract of leaves; Figure S5: EI-MS spectrum of fucosterol; Figure S6: ^1^H-NMR spectrum of fucosterol (CDCl_3_, 400 MHz)**;** Figure S7: APT spectrum of fucosterol (CDCl_3_, 100 MHz); Figure S8: EI-MS spectrum of stigmasterol; Figure S9: ^1^H-NMR spectrum of stigmasterol (CDCl_3_, 500 MHz); Figure S10: ^13^C-NMR spectrum of stigmasterol (CDCl_3_, 125 MHz); Figure S11: EI-MS spectrum of ursolic acid; Figure S12: ^1^H-NMR spectrum of ursolic acid (DMSO, 500 MHz); Figure S13: ^13^C-NMR spectrum of ursolic acid (DMSO, 125 MHz); Figure S14: Investigation of *G. thunbergia* methanol extract of leaves n-Butanol fraction; Figure S15: ESI-MS (negative mode) spectrum of rutin; Figure S16: ^1^H-NMR spectrum of rutin (DMSO, 400 MHz); Figure S17: ^13^C-NMR spectrum of rutin (DMSO, 100 MHz); Figure S18: HMBC spectrum of rutin (DMSO).

## Sample Availability

Samples of the compounds are not available from the authors.
