# Supplementary material for: Elucidation of Natural Components of Gardenia thunbergia Thunb. Leaves: Effect of Methanol Extract and Rutin on Non-Alcoholic Fatty Liver Disease
Source: Molecules. 2023 Jan 16;28(2):879. doi: 10.3390/molecules28020879 (PMC9866290; doi:10.3390/molecules28020879)
Supplement: Supplementary file 1 [file molecules-28-00879-s001.zip › molecules-2128006-supplementary.pdf]

molecules

Supplementary Materials

Elucidation of Natural Components of *Gardenia thunbergia* Thunb. Leaves: Effect of Methanol Extract and Rutin on Non-Alcoholic Fatty Liver Disease

Eman M. EL-Shial [1,2], Amal Kabbash [1], Mona El-Aasr [1], Ola. A. El-Feky [3] and Suzy A. El-Sherbeni [1,*]

1 Department of Pharmacognosy, Faculty of Pharmacy, Tanta University, Tanta 31527, Egypt
2 Department of Pharmacognosy, Faculty of Pharmacy, Horus University, New Damietta 34518, Egypt
3 Department of Biochemistry, Faculty of Pharmacy, Tanta University, Tanta 31527, Egypt
* Correspondence: suzy.elsherbini@pharm.tanta.edu.eg

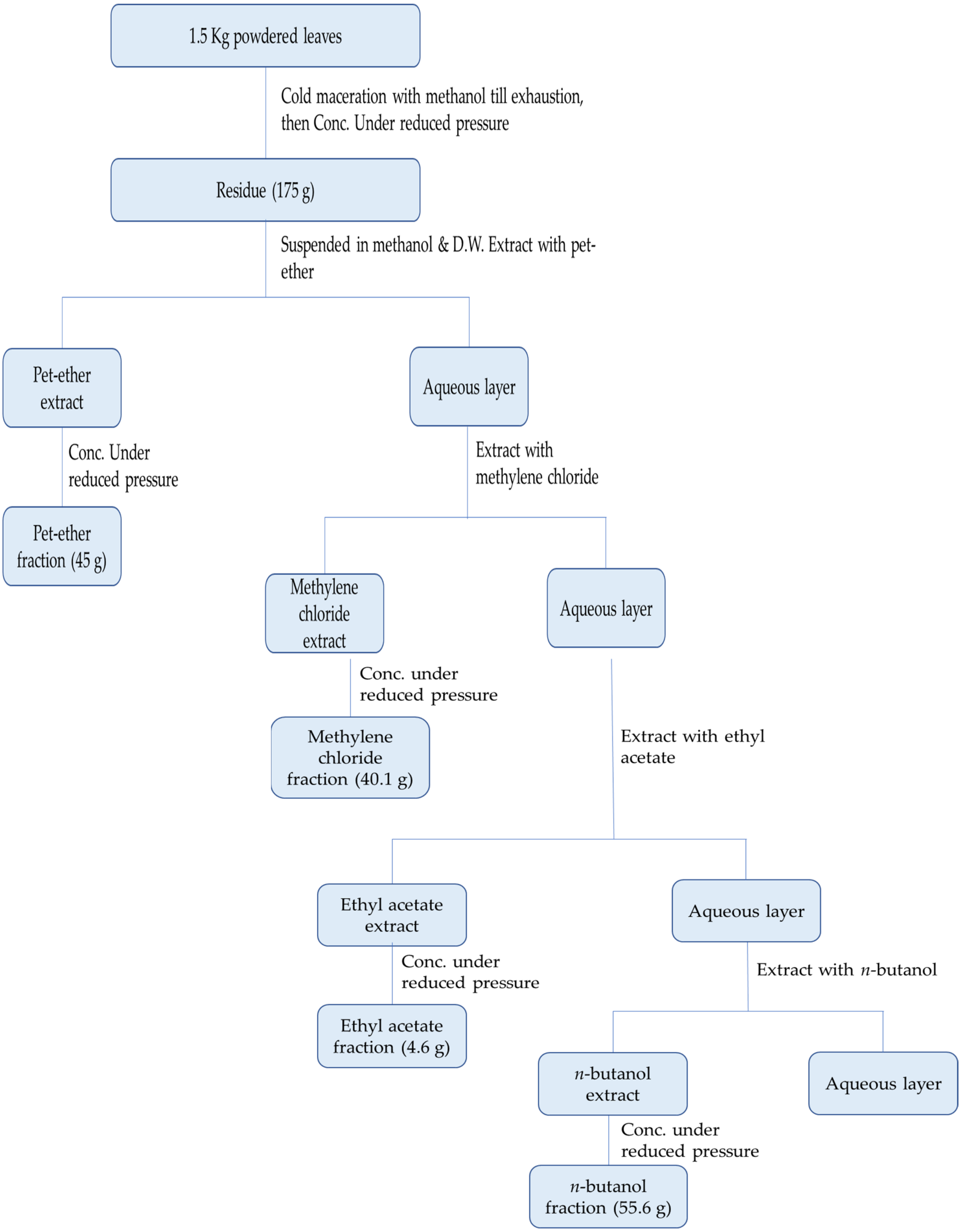

Figure S1. Extraction of *G. thunbergia* methanol extract of leaves and fractionation.

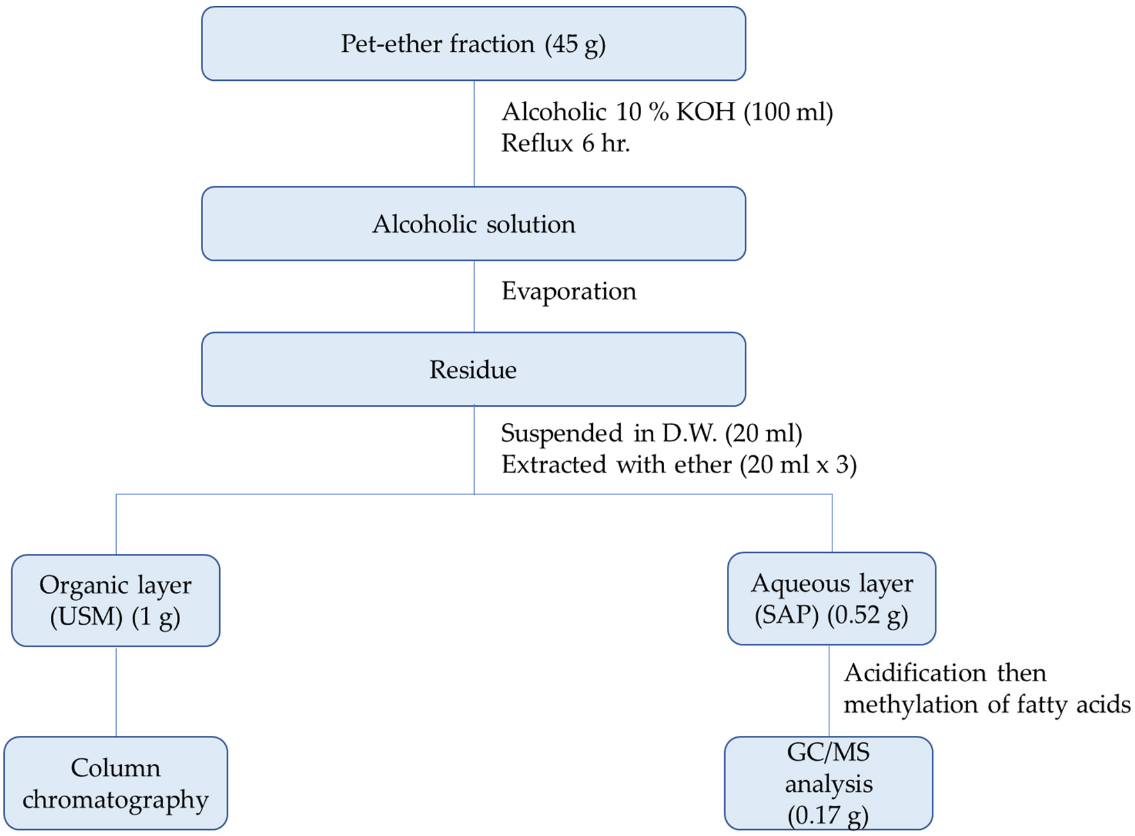

Figure S2: Investigation of petroleum ether extract of *G. thunbergia* methanol extract of leaves

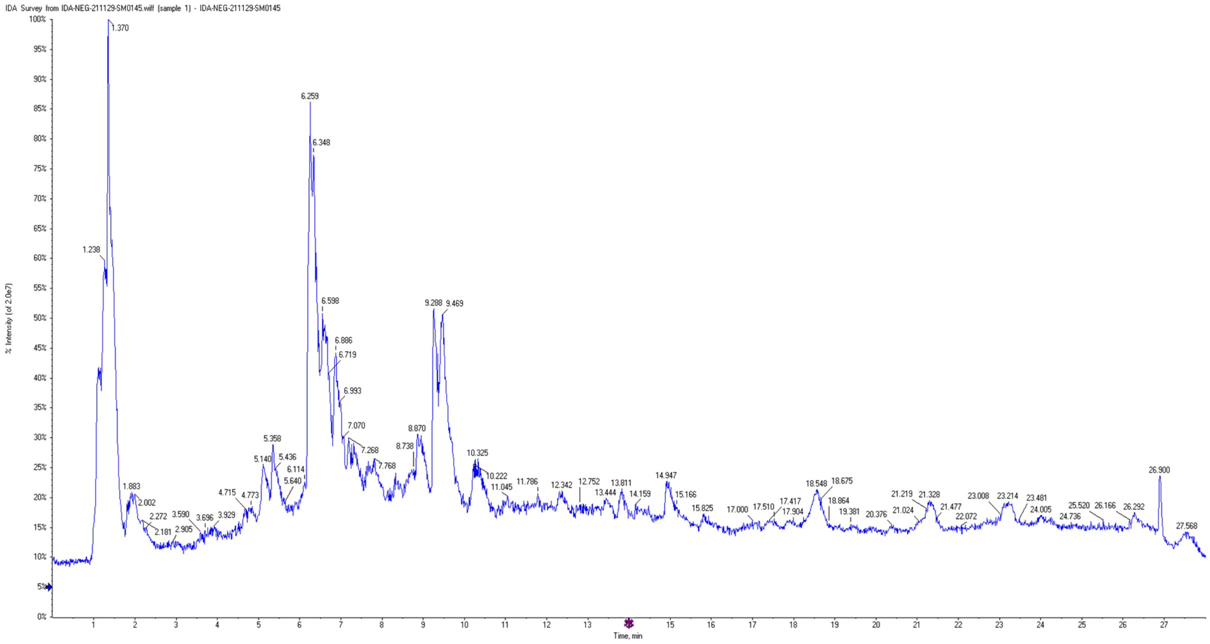

Figure S3: The total ion chromatogram (TIC) of *G. thunbergia* methanol extract of leaves (negative mode)

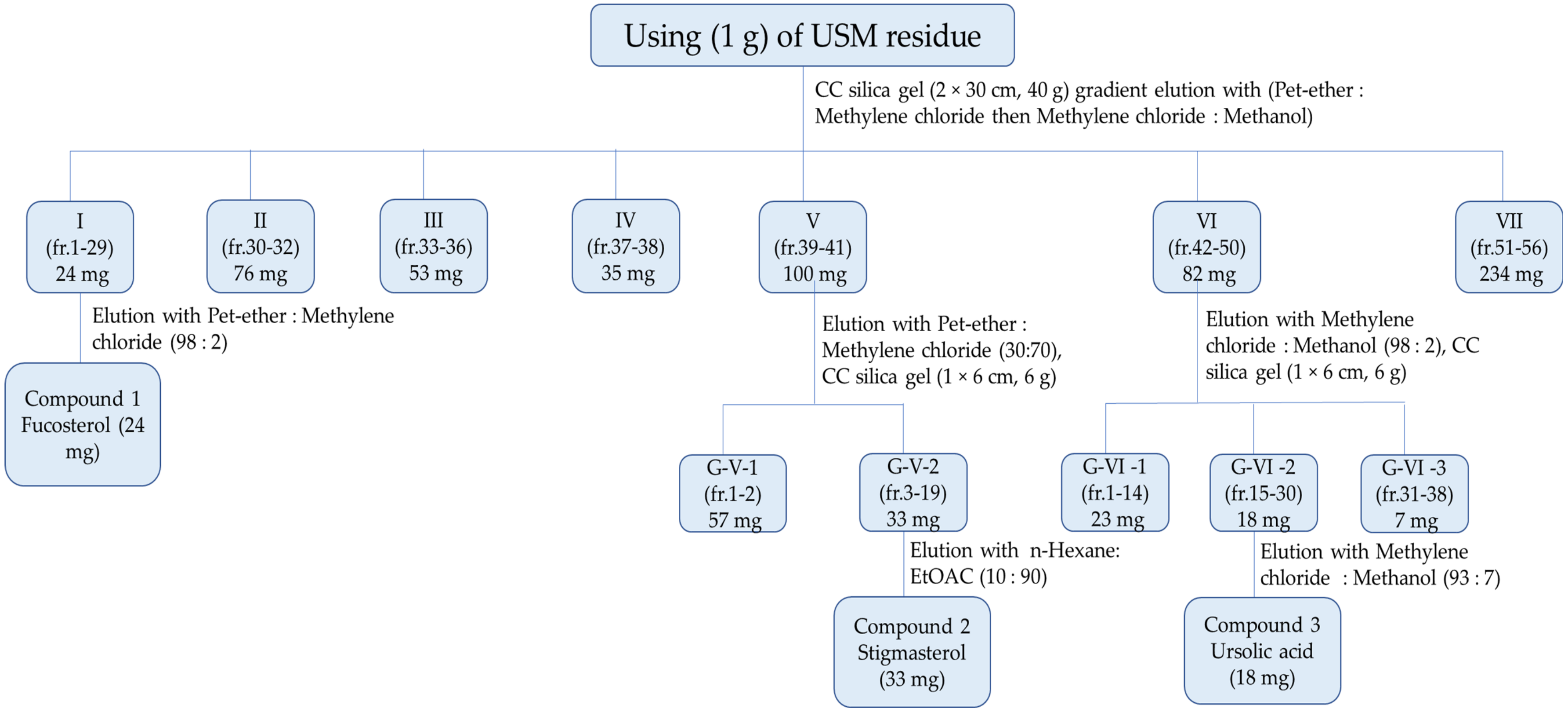

Figure S4: Investigation of the unsaponifiable matter (USM) of *G. thunbergia* methanol extract of leaves

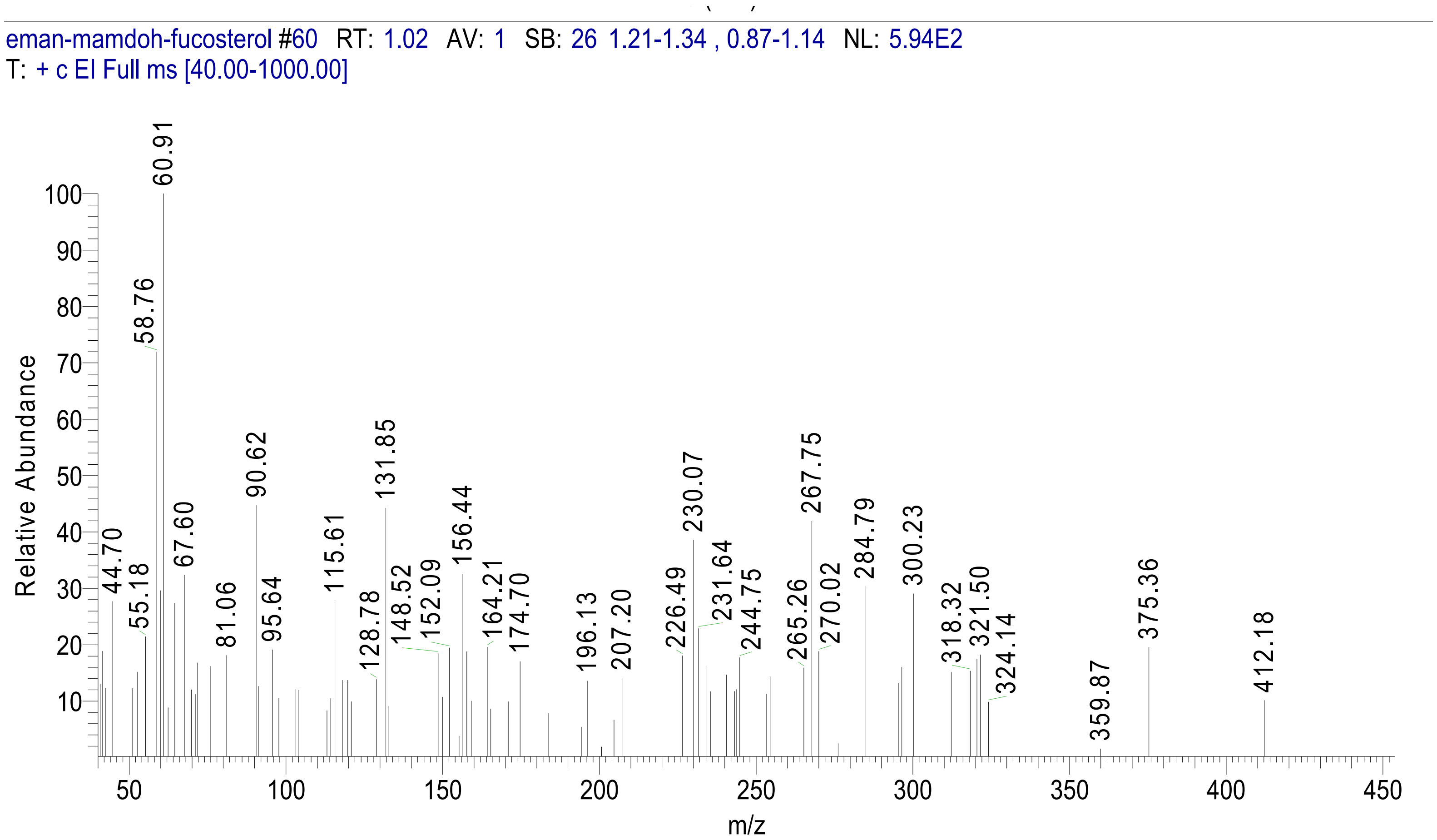

Figure S5: EI-MS spectrum of fucosterol

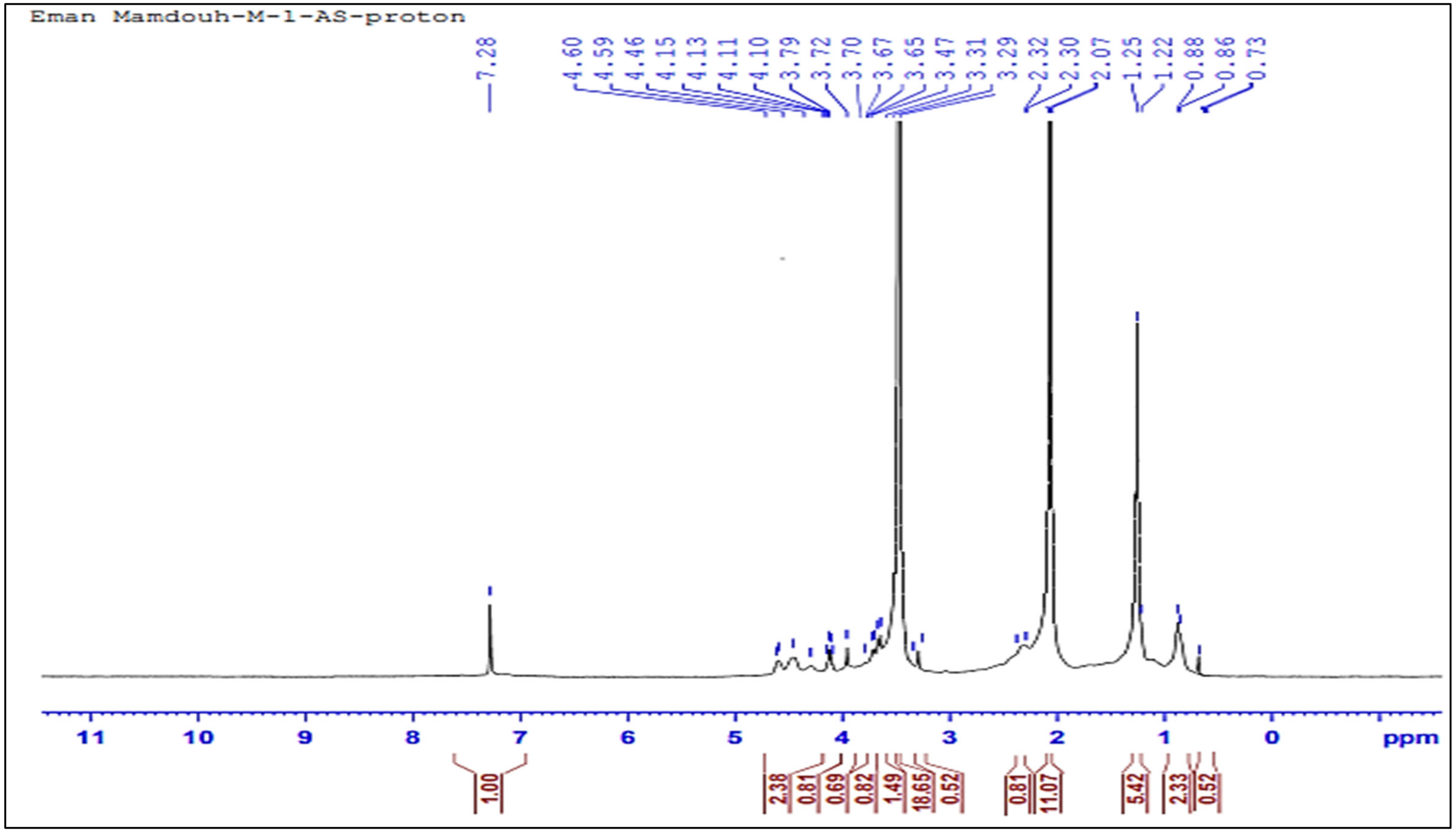

Figure S6: ^{1}H-NMR spectrum of fucosterol ($CDCl_3$, 400 MHz)

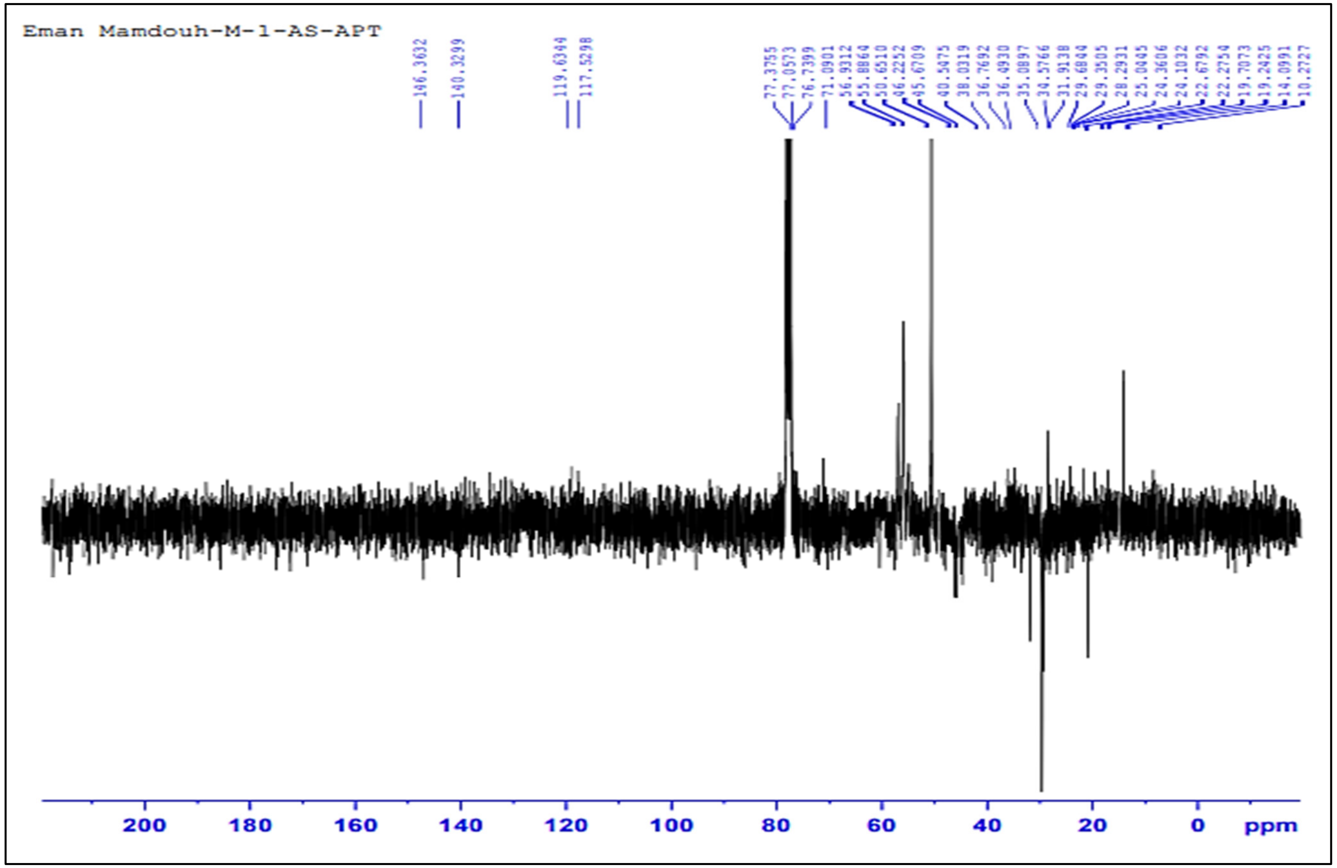

Figure S7: APT-NMR spectrum of fucosterol ($CDCl_3$, 100 MHz)

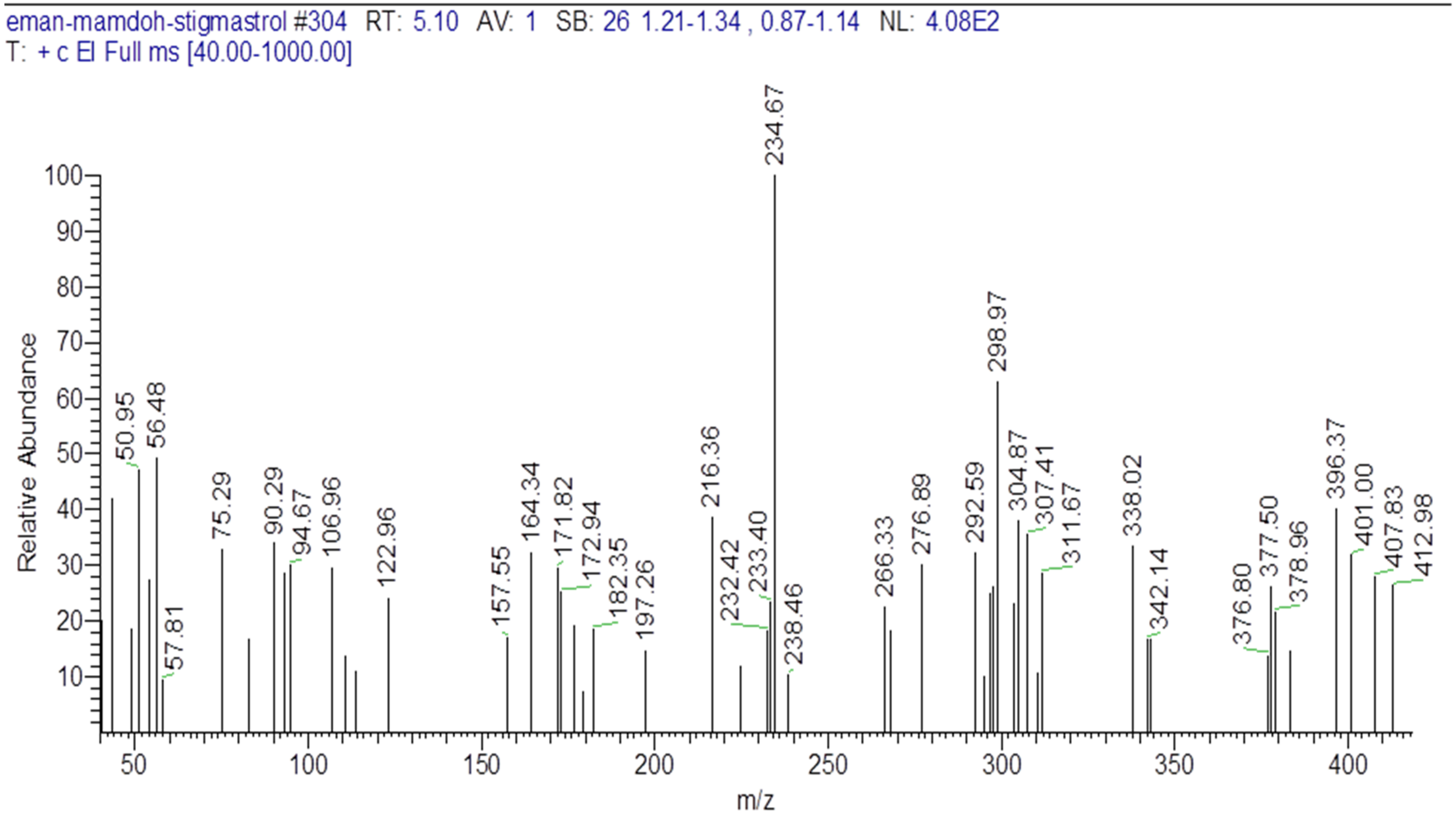

Figure S8: EI-MS spectrum of stigmasterol

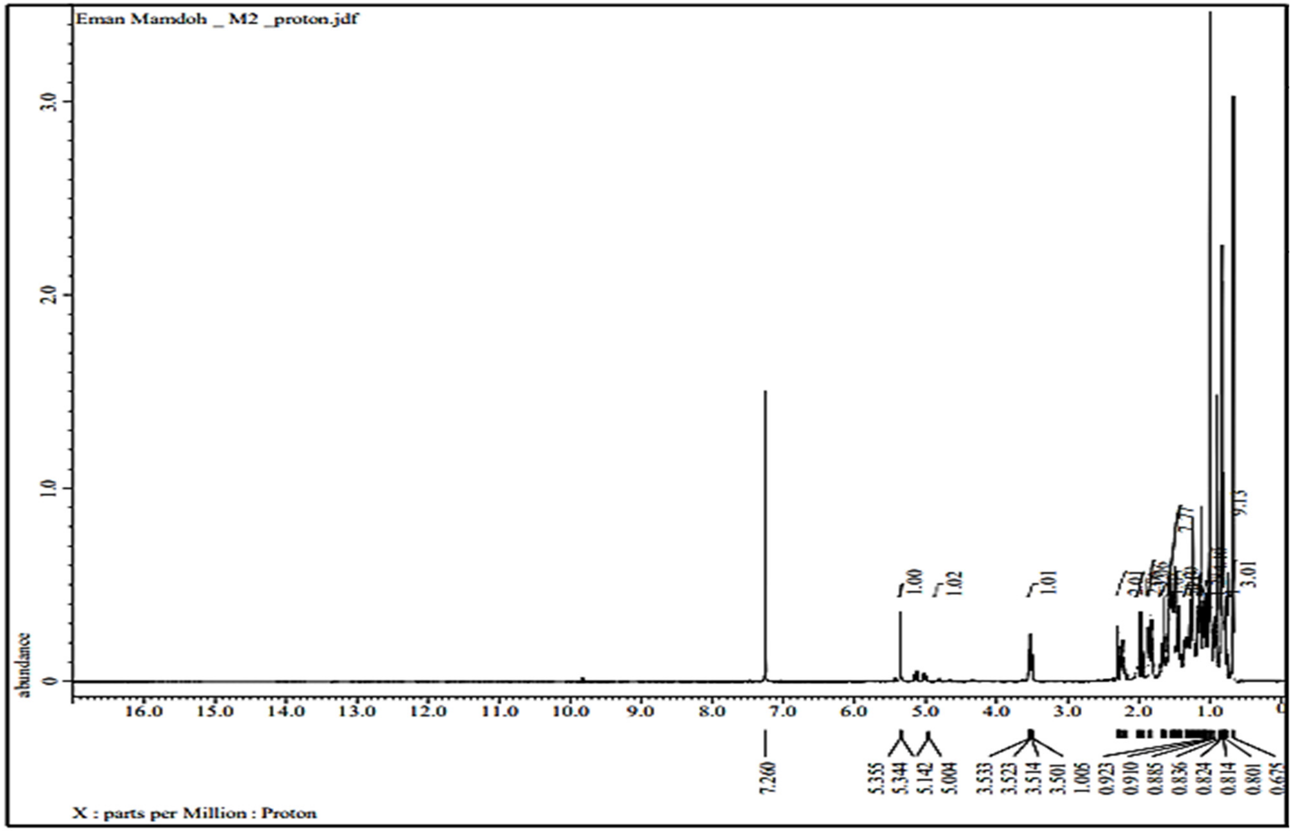

Figure S9: ^{1}H-NMR spectrum of stigmasterol ($CDCl_3$, 500 MHz)

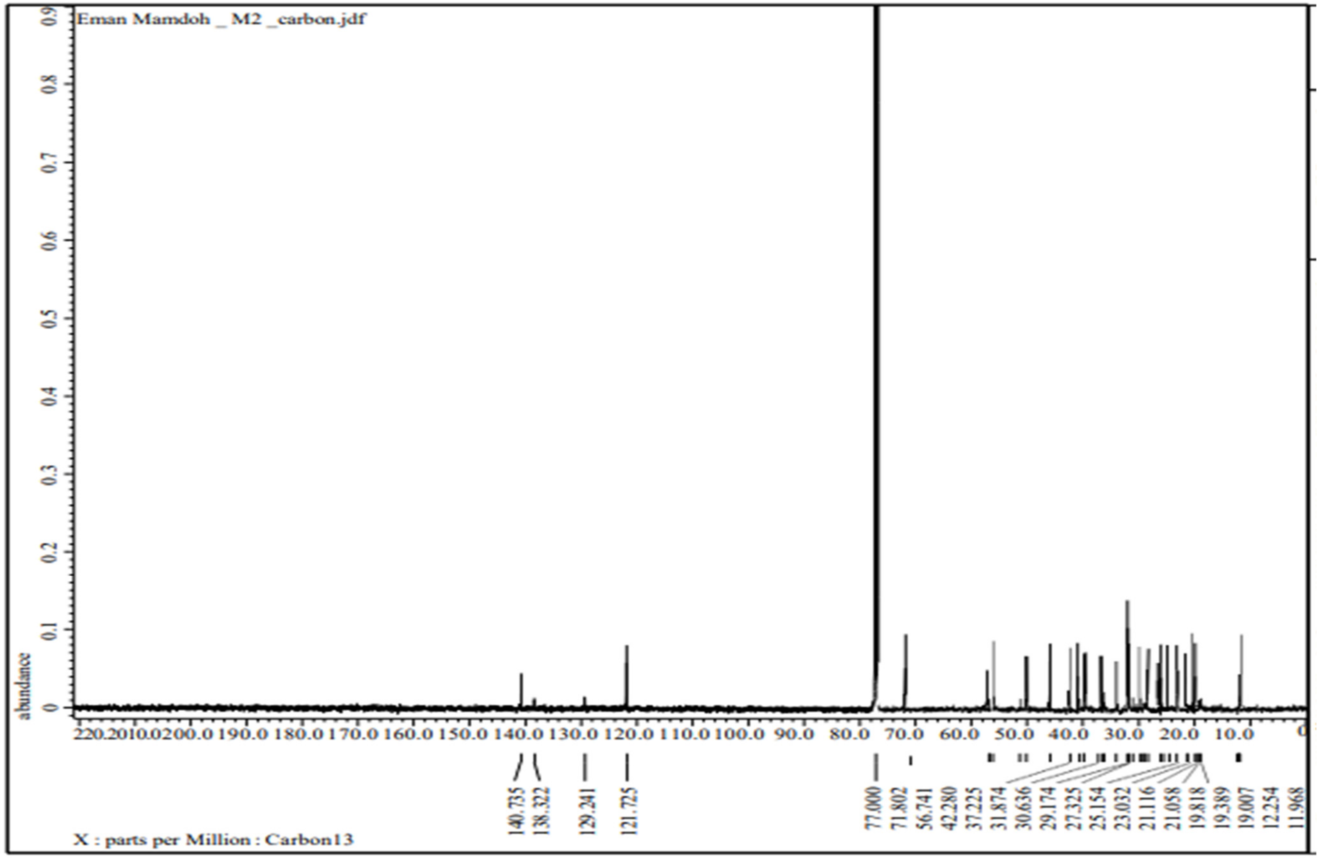

Figure S10: ^{13}C-NMR spectrum of stigmasterol ($CDCl_3$, 125 MHz)

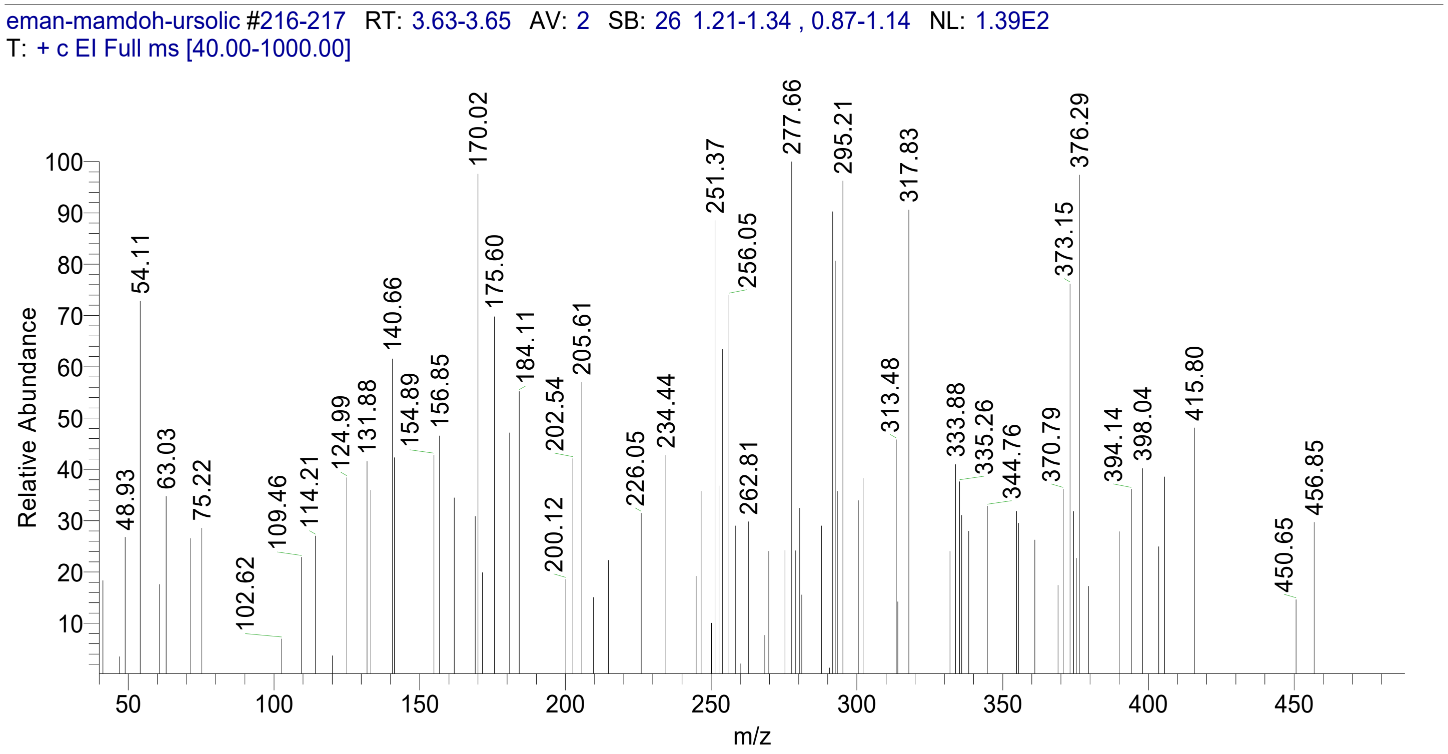

Figure S11: EI-MS spectrum of ursolic acid

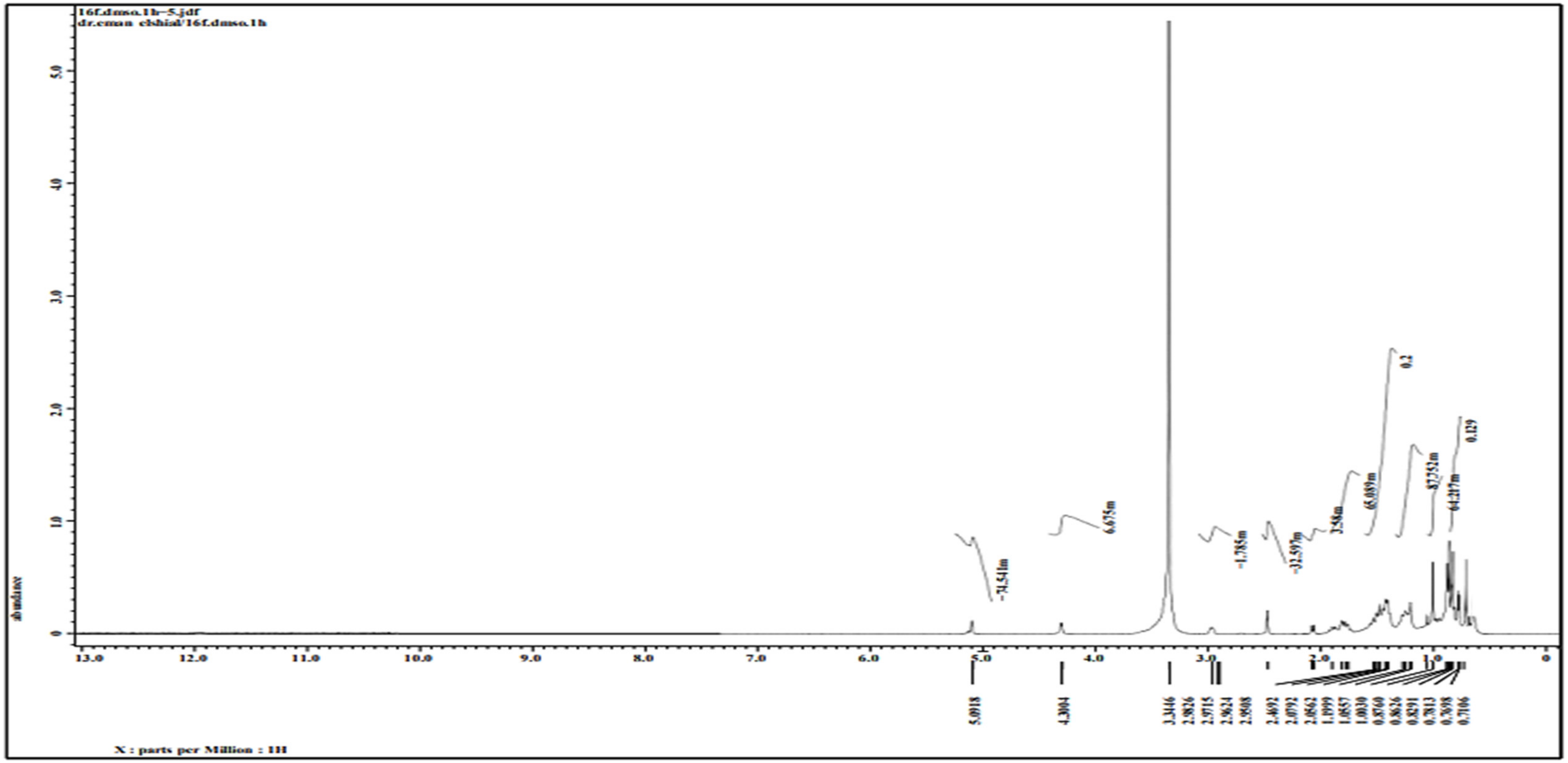

Figure S12: ^{1}H-NMR spectrum of ursolic acid (DMSO, 500 MHz)

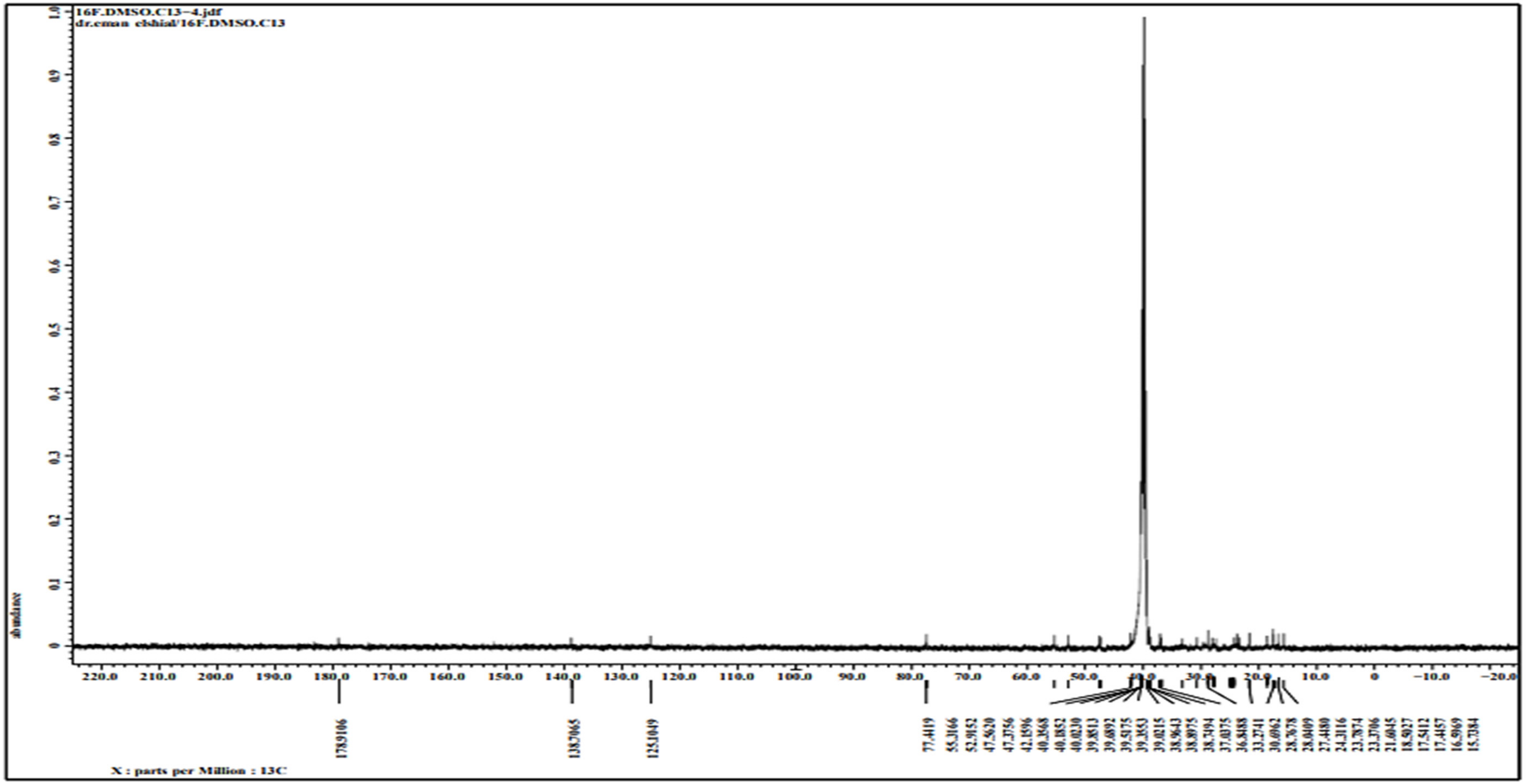

Figure S13: ^{13}C-NMR spectrum of ursolic acid (DMSO, 125 MHz)

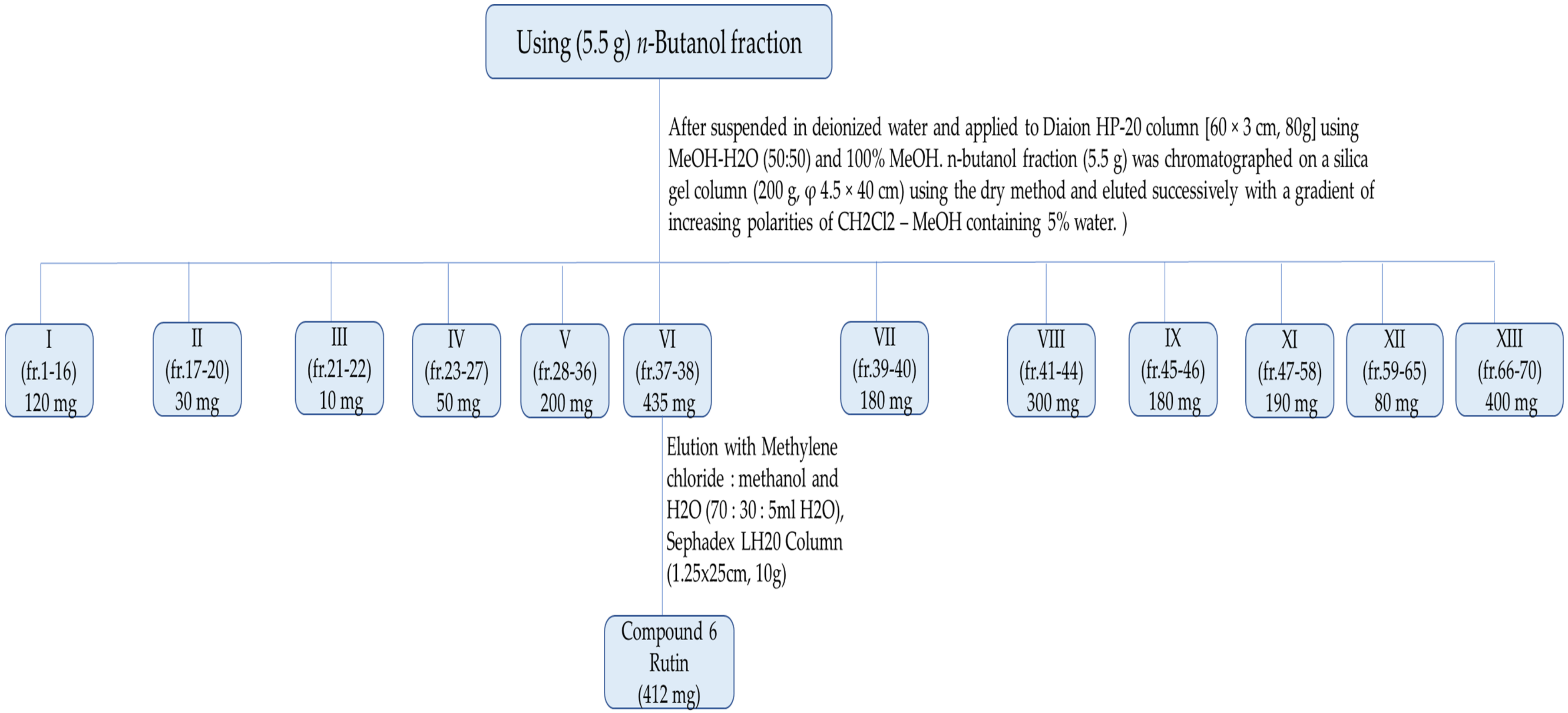

Figure S14: Investigation of *G. thunbergia* methanol extract of leaves *n*-Butanol fraction

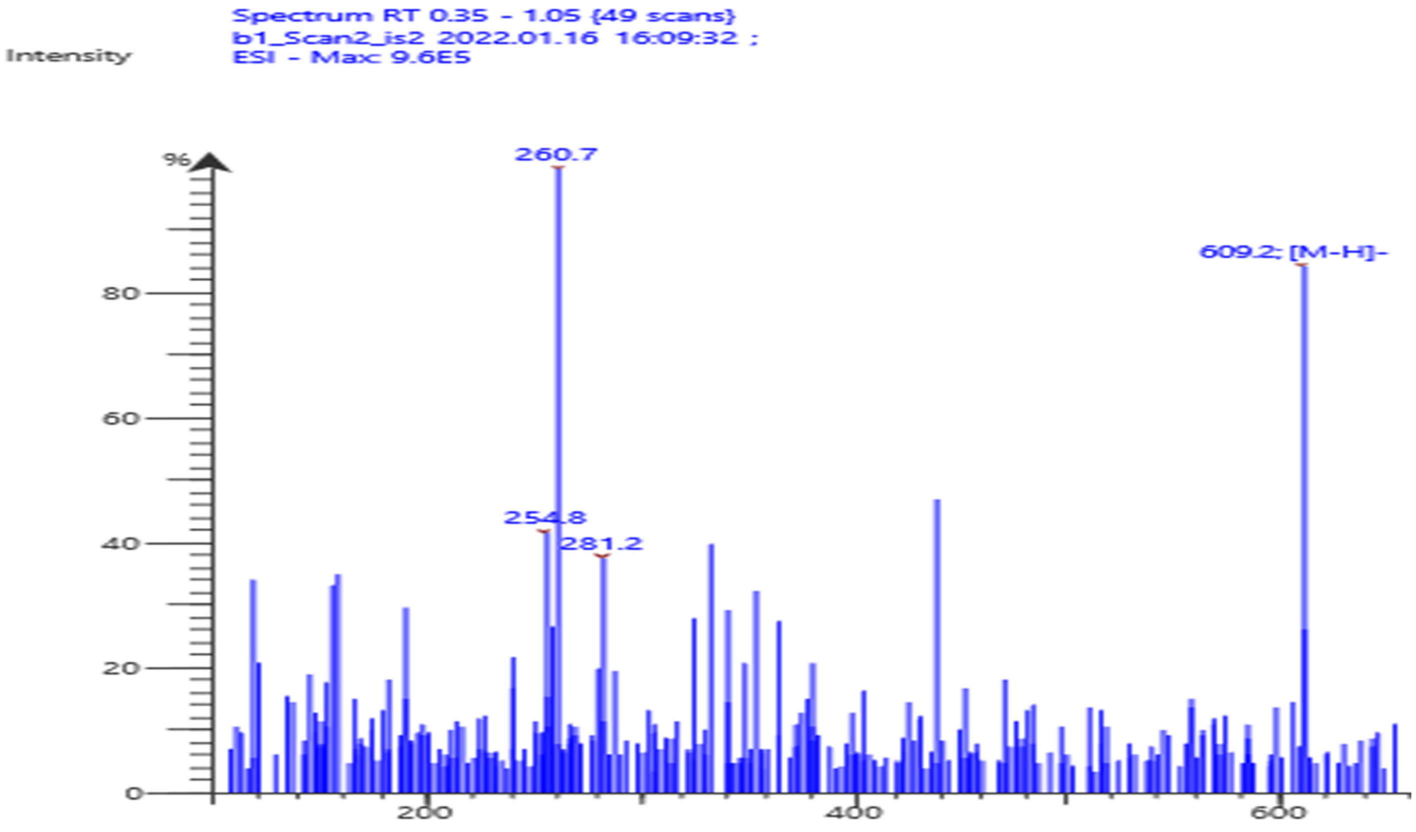

Figure S15: ESI-MS (negative mode) spectrum of rutin

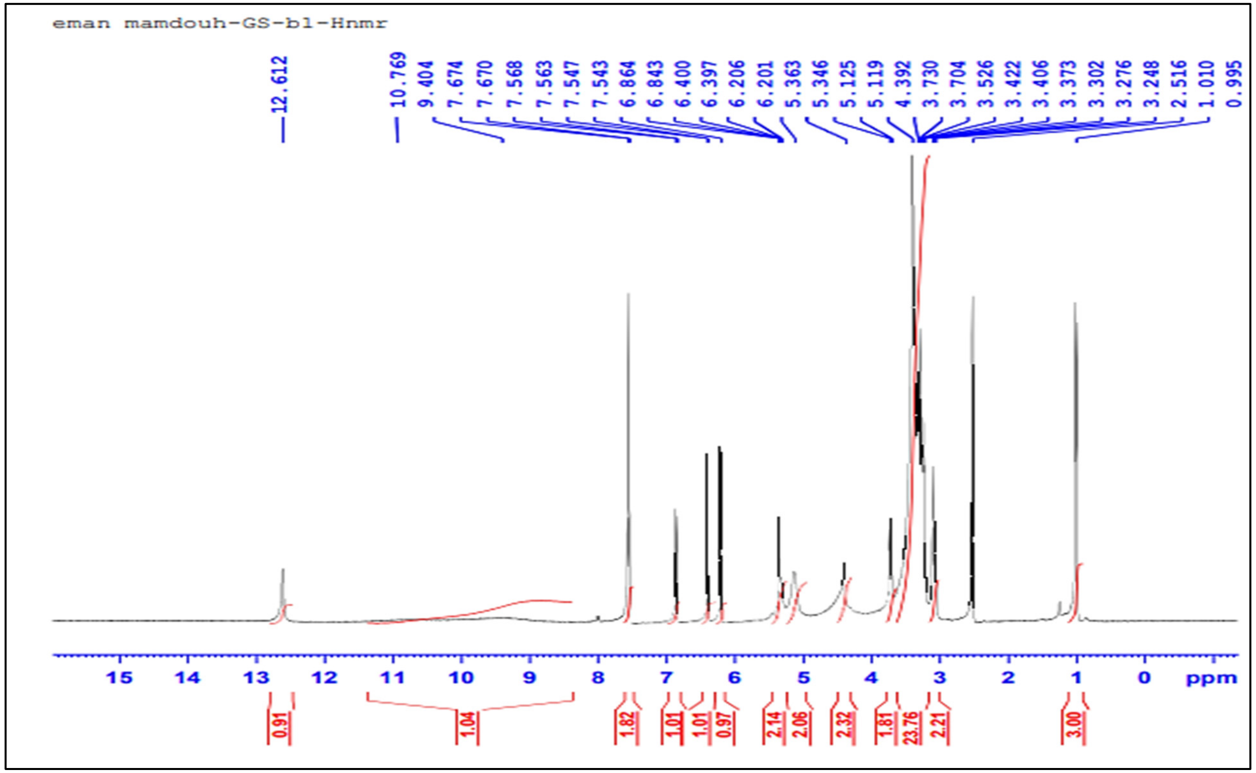

Figure S16: ^{1}H-NMR spectrum of rutin (DMSO, 400 MHz)

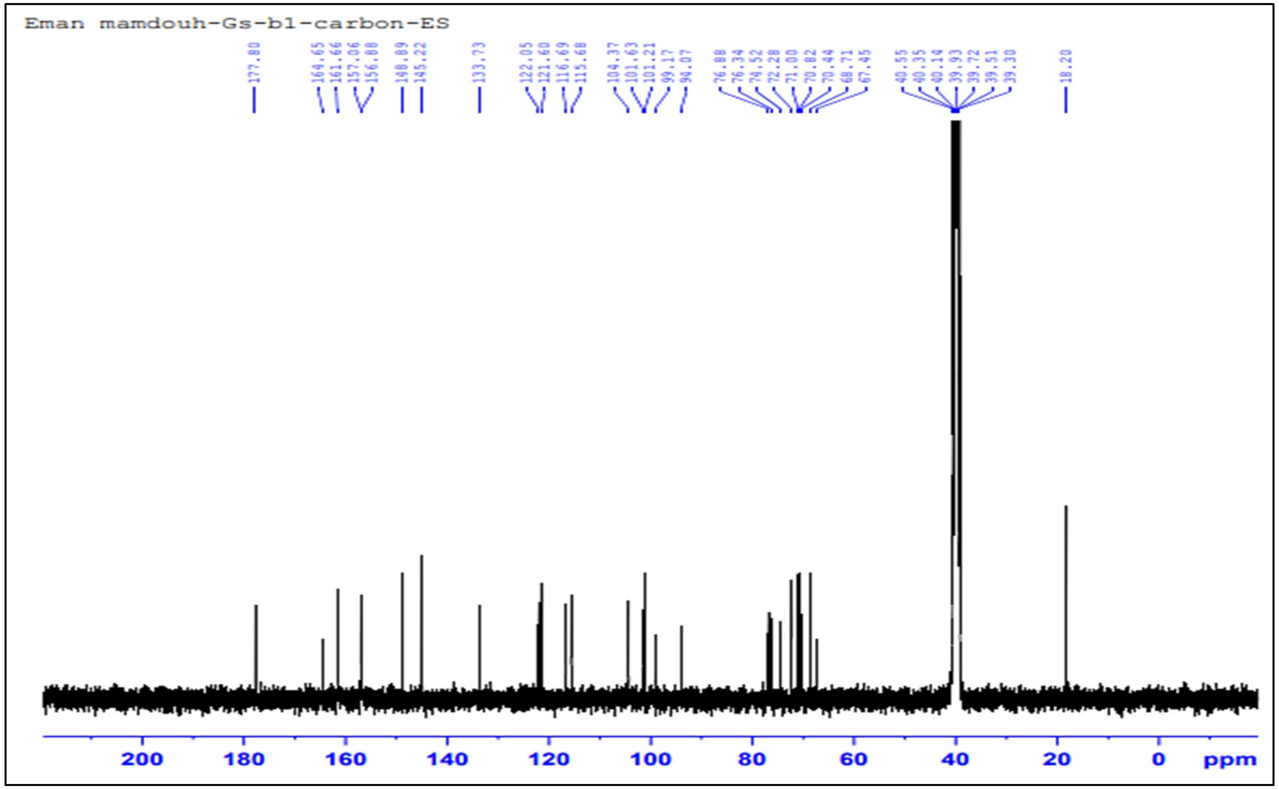

Figure S17: ^{13}C-NMR spectrum of rutin (DMSO, 100 MHz)

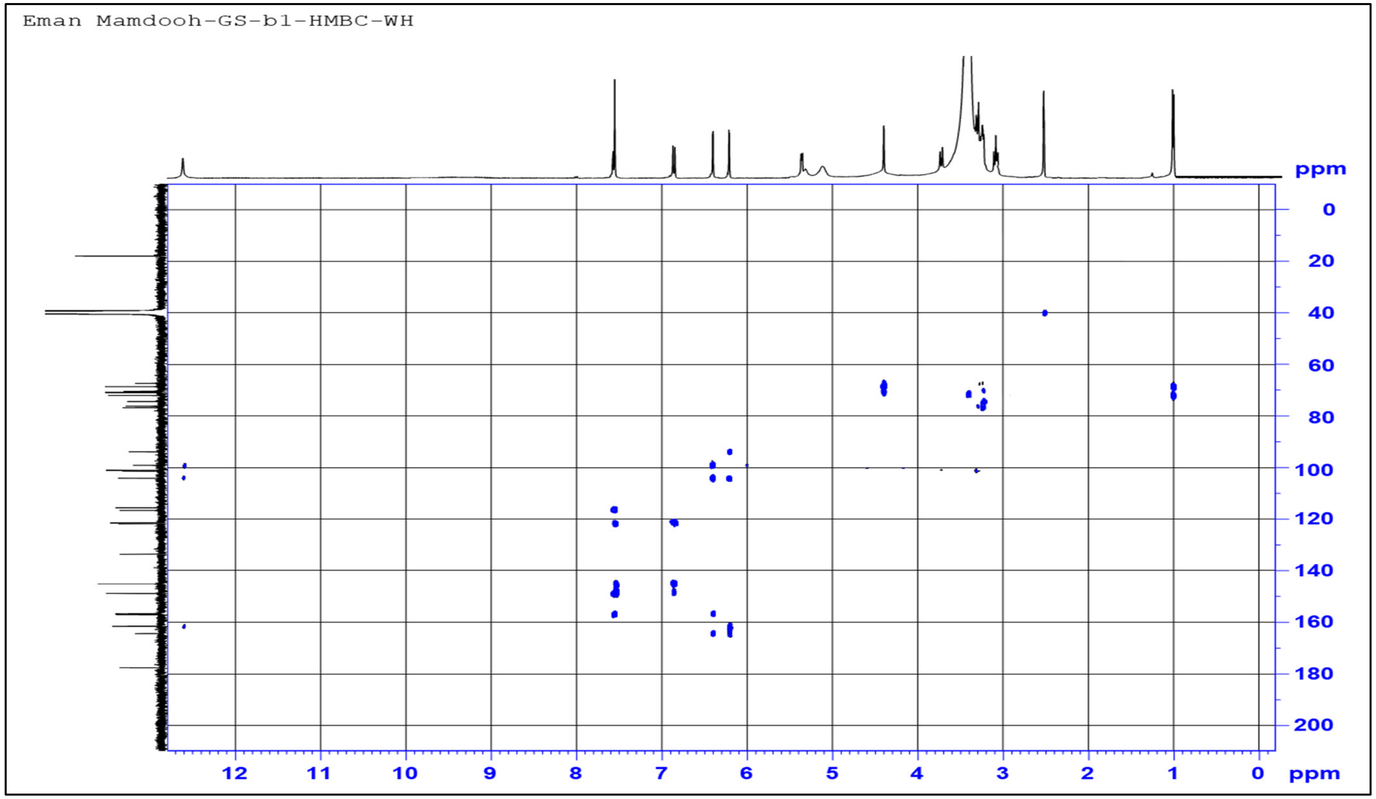

Figure S18: HMBC spectrum of rutin (DMSO)

Compound (1):

^{1}H-NMR (chemical shift δ in ppm) (400 MHz, $CDCl_3$): 4.60 (1H, m, H-6), 4.15 (1H, m, H-28), 3.47 (1H, m, H-3), 3.67 (1H, m, H-25), 3.29 (3H, d, *J* = 8 Hz, H-29), 2.30 (d, *J* = 8 Hz, H-21), 2.07 (3H, s, H-19), 1.22 (3H, d, *J* = 12 Hz, H-27), 0.88 (3H, d, *J* = 8 Hz, H-26), 0.73 (3H, s, H-18). APT-NMR (100 MHz, $CDCl_3$): 38.03 (C-1), 29.35 (C-2), 71.09 (C-3), 45.67 (C-4), 140.32 (C-5), 119.63 (C-6), 31.91 (C-7), 29.68 (C-8), 50.65 (C-9), 36.76 (C-10), 22.27 (C-11), 40.54 (C-12), 46.22(C-13), 56.93 (C-14), 24.36 (C-15), 28.29 (C-16), 55.88 (C-17), 10.27 (C-18), 19.70 (C-19), 36.49 (C-20), 19.24 (C-21), 35.08 (C-22), 25.04 (C-23), 146.36 (C-24), 34.57 (C-25), 22.67 (C-26), 24.10 (C27), 117.52(C-28), 14.09 (C-29); EI-MS *m/z*: 412.18 $[M]^+$.

Compound (2):

^{1}H-NMR (chemical shift δ in ppm) (500 MHz, $CDCl_3$): 5.35 (1H, m, H-6), 5.14 (2H, m, H-23), 5.00 (2H, m, H-22), 3.51(1H, m, H-3), 0.67 (3H, s, H-18), 0.92 (3H, d, *J* = 6.5 Hz, H-21), 0.88 (3H, t, H-29),0.83 (3H, d, *J* = 6 Hz, H-27), 0.81 (3H, d, *J* = 6.5 Hz, H-26), 1.00 (3H, s, H-19). ^{13}C-NMR (125 MHz, $CDCl_3$): 37.22 (C-1), 31.87 (C-2), 71.80 (C-3), 42.28 (C-4), 140.73 (C-5), 121.72 (C-6), 30.63 (C-7), 30.63 (C-8), 50.05 (C-9), 34.92 (C-10), 21.05 (C-11), 39.93 (C-12), 42.28 (C-13), 56.74 (C-14), 23.03 (C-15), 27.32 (C-16), 56.17 (C-17), 12.25 (C-18), 19.00 (C-19), 40.58 (C-20), 21.11 (C-21), 138.32 (C-22), 129.24 (C-23), 46.15 (C-24), 29.17 (C-25), 19.38 (C-26), 19.81 (C-27), 25.15 (C-28), 11.96 (C-29); EI-MS *m/z*: 412.98 $[M]^+$.

Compound (3):

^{1}H-NMR (chemical shift δ in ppm) (500 MHz, DMSO): 5.09 (1H, s, H-12), 4.30 (1H,s, OH), 2.97 (1H, m, H-3), 2.46 (1H, d, *J* = 11.3 Hz, H-18), 1.19 (3H, s, H-23), 1.05 (3H, s, H-27), 1.00 (3H, s, H-26), 0.82 (3H, s, H-24), 0.86 (3H, d, *J* = 6.7 Hz, H-30), 0.76 (3H, d, *J* = 5.75 Hz, H-29), 0.71 (3H, s, H-25). ^{13}C-NMR (125 MHz, DMSO): 38.89 (C-1), 28.04 (C-2), 77.44 (C-3), 38.74 (C-4), 55.31 (C-5), 18.50 (C-6), 33.27 (C-7), 39.02 (C-8), 47.56 (C-9), 37.03 (C-10), 23.37 (C-11), 125.10 (C-12), 138.70 (C-13), 42.15 (C-14), 27.44 (C-15), 24.31 (C-16), 47.37 (C-17), 52.91 (C-18), 38.96 (C-19), 38.96 (C-20), 30.69 (C-21), 36.84 (C-22), 28.04 (C-23), 15.73 (C-24), 16.59 (C-25), 17.44 (C-26), 23.78 (C27), 178.91(C-28), 17.54 (C-29), 21.60 (C-30); EI-MS *m/z* = 456.85 [1].

Compound (4):

^{1}H-NMR (chemical shift δ in ppm) (400 MHz, DMSO): δ 7.56 (d, *J* = 2 Hz, H-6′), 7.54 (s, H-2′), 6.84 (d, *J* = 8.4 Hz, H-5′), 6.40 (d, *J* = 1.2 Hz, H-8), 6.20 (d, *J* = 2 Hz, H-6), 5.35 (d, *J* = 6.8 Hz, H-glc-1), 3.37 (m, H-glc-2), 3.73 (d, *J* = 10.4 Hz, H-glc-6), 4.39 (s, H-rha-1), 3.37 (m, H-rha-2), 3.24 (m, H-rha-3), 3.37 (m, H-rha-4), 3.24 (m, H-rha-5), 1.01 (d, *J* = 6 Hz, H-rha-6).^{13}C-NMR (100 MHz, DMSO): 156.88 (C-2), 133.73 (C-3), 177.81 (C-4), 161.66 (C-5), 99.17 (C-6), 164.65 (C-7), 94.07 (C-8), 157.06 (C-9), 104.37 (C-10), 122.05 (C-1′), 116.70 (C-2′), 145.22 (C-3′), 148.89 (C-4′), 115.69 (C-5′), 121.61 (C-6′), 101.63 (C-glc-1), 74.52 (glc-2), 76.88 (glc-3), 68.71 (glc-4), 76.35 (glc-5), 67.45 (glc-6), 101.21 (C-rha-1), 71.00 (rha-2), 70.44 (rha-3), 70.82 (rha-4), 72.28 (rha-5), 18.21 (rha-6); ESI-MS (negative mode) $[M-H]^-$ at *m/z*: 609.2.

Quantitative real-time polymerase chain reaction (qRT-PCR) for liver tissue

Primers sequences designed by Primer 3 plus Program (version 2.0). Primer sequences (Willowfort. Co., UK) were as follows**: CYP2E1 gene** forward primer: 5′-TTC CCT AAG TAT CCT CCG TGA- 3′, reverse primer: 5′-CGT AAT CGA AGC GTT TGT TG-3′, **c-JNK1 gene** forward primer: 5′-GCC ATT CTG GTA GAG GAA GTT TCT C-3′, reverse primer: 5′-CGC CAG TCC AAA ATC AAG AAT C-3′, **iNOS** gene forward primer: 5′-TGA CGC CAA ACA TGA CTT CAG-3′, reverse primer: 5′-GCC ATC GGG CAT CTG GTA-3′, and **ß-actin (reference gene)** forward primer: 5′-CAT TGC TGA CAG GAT GCA GAA GG-3′, reverse primer: 5′-TGC TGG AAG GTG GAC AGT GAG G-3′.
